# Increased relative abundance of *Alistipes* and *Sellimonas* is related to stage 2 and 3 sleep duration

**DOI:** 10.3389/frsle.2025.1478129

**Published:** 2025-05-30

**Authors:** Hiroyuki Sasaki, Hirofumi Masutomi, Katsuyuki Ishihara

**Affiliations:** Research & Development Division, Calbee, Inc., Utsunomiya, Tochigi, Japan

**Keywords:** brain-gut-interaction, microbiota, sleep quality, non-REM sleep, causality search

## Abstract

Sleep is important for maintaining body homeostasis, and lack of sleep or poor sleep quality increases the risk of various diseases. In recent years, it has been shown that there is an interaction between the gut microbiota and brain function, known as the brain-gut interaction. Although several studies have examined the relationship between gut microbiota and sleep, most of them rely on subjective indicators, and there are few reports using objective sleep measurements. Therefore, the aim of this study was to clarify the relationship between gut microbiota and sleep using various statistical analysis methods based on data obtained from the database. First, we obtained data from the *Sukoyaka* Health Survey, and performed hierarchical clustering analysis of the electroencephalogram (EEG)-derived sleep parameters. We examined the intestinal bacteria that differed significantly among clusters, and the relationship between intestinal bacteria and EEG-derived sleep parameters using multiple regression analysis and causal search. Multiple regression analysis and causal search suggested a relationship between increased *Sellimonas* levels and increased non-rapid eye-movement (non-REM) sleep stage 2, and increased *Alistipes* levels and increased non-REM sleep stage 3. The results of the causal search indicated that *Alistipes* and *Sellimonas* may influence the duration of non-REM sleep stage.

## 1 Introduction

Sleep takes almost one-third of the day and functions as homeostasis to recover from body fatigue and brain functions (Garbarino et al., 2021). For example, in terms of physical function, sleep is associated with the regulation of nearly every system in the body, including the autonomic nervous (Tavares et al., 2021), cardiovascular (Covassin and Singh, 2016), immune (Irwin, 2019) and metabolic systems (Magee and Hale, 2012), and in terms of brain function, sleep plays an important role in cognitive performance (Van Dongen et al., 2003), memory consolidation (Tononi and Cirelli, 2014), and mood regulation (Lieberman et al., 2005).

Brain activity changes in various ways, even during sleep. Sleep is broadly classified into rapid eye movement (REM) and non-REM, based on electroencephalogram (EEG). Non-REM sleep is further divided into the stages 1 to 4 (N1, N2, N3, and N4 respectively) (Hori et al., 2001; Ackermann and Rasch, 2014).

In recent years, sleep deprivation (sleep loss) has attracted attention as a global health issue. Sleep disorders have become a global public health problem, affecting approximately 15%−30% of adults and placing a significant burden on quality of life (Zammit et al., 1999; Karna et al., 2024). Sleep deprivation has been reported to be associated with increased risk of various health risks, including cardiovascular disease, diabetes, metabolic syndrome, and depression (Reutrakul and Van Cauter, 2018; Tobaldini et al., 2019; Agrawal et al., 2022). Not only the duration of sleep, but also the duration of REM and non-REM sleep is important. For example, it has been reported that a ratio of REM sleep to total sleep time of < 15% is associated with an increased risk of death from cardiovascular diseases and other causes (Leary et al., 2020). The percentage of non-REM sleep stage 3 has also been reported to be correlated with daytime sleepiness, exercise performance, and problem-solving performance, and is important for overall daytime activity (Dijk, 2009; McCarter et al., 2022). Additionally, it has been reported that a lower ratio of non-REM sleep stage 3 can lead to anxiety and depression (Motomura et al., 2013).

During non-REM sleep, brain and autonomic nervous system activities decrease, including heart rate, respiratory rate, and blood pressure (Pagani et al., 1986). One of the important restorative functions of sleep is related to tissue repair, particularly in the musculoskeletal and immune systems. During slow-wave sleep (non-REM sleep stage 3), the body increases the production of growth hormone, which is an important mediator of tissue repair and cell regeneration (Redwine et al., 2000). Growth hormone stimulates protein synthesis and cell division, promotes the repair of damaged muscle fibers and accelerates wound healing (Dioufa et al., 2010; Chikani and Ho, 2014). Sleep is also associated with an increase in the production of cytokines, signaling molecules that play an important role in regulating immune responses and inflammation (Redwine et al., 2000). Insufficient sleep can lead to reduced cytokine levels, which can impair the body's ability to fight infection and recover from inflammation (Veler, 2023).

Memory consolidation is one of the best studied functions of sleep and involves the stabilization and integration of newly acquired information into long-term memory. This process occurs primarily during slow-wave sleep (non-REM sleep stage 3 and 4). During this stage, the brain engages in “replay” activity, reactivating the patterns of activity experienced during waking by neurons in the hippocampus, which is responsible for short-term memory. This reactivation strengthens the synaptic connections between the hippocampus and the neocortex, allowing memories to be transferred to more permanent memory areas (Genzel and Battaglia, 2017; Frazer et al., 2021; Reyes-Resina et al., 2021). In contrast, REM sleep is accompanied by the loss of skeletal muscle tone and a decrease in thermoregulation, but the brain waves are very similar to those during wakefulness (Dong et al., 2022). Therefore, REM sleep is considered important for intellectual development (Maquet et al., 1996; Nofzinger et al., 1997). Sleep helps to balance synaptic strength through a process known as synaptic homeostasis. During wakefulness, as the brain processes and responds to environmental stimuli, synapses are often strengthened. However, this strengthening consumes energy and neural capacity, and the circuits can become “overloaded” (Tononi and Cirelli, 2006). During sleep, and particularly during slow-wave sleep, the brain selectively weakens less important synapses while maintaining important connections (a process called synaptic downscaling) (Blanco et al., 2015; Liu et al., 2024). This pruning allows the brain to maintain its efficiency and be ready to process new information the next day. One of the most remarkable discoveries in recent years is the glymphatic system, a network that promotes the removal of neurotoxic waste products from the brain during sleep (Xie et al., 2013; Rasmussen et al., 2018). This system is mainly active during non-REM sleep and relies on the exchange of cerebrospinal fluid and interstitial fluid to flush out metabolic by-products such as amyloid beta protein and tau protein, substances associated with neurodegenerative diseases such as Alzheimer's (Reddy and van der Werf, 2020; Shirolapov et al., 2024). Because of these various processes, sleep is important for the recovery of physical and brain function.

Sleep is strongly influenced by the internal and external environments, including circadian rhythms, light environment, and diet, one of which is the intestinal microbiota (Ogawa et al., 2020). The mammalian intestinal microbiota comprises approximately 100 trillion bacterial cells. A symbiotic relationship has been established between the intestinal microbiota and hosts. The intestinal microbiota grow by utilizing indigestible nutrients that cannot be digested or absorbed by the host, and the host utilizes the metabolites produced by the fermentation and degradation of indigestible nutrients by the intestinal microbiota to regulate physiological functions (Koh et al., 2016; Marchesi et al., 2016; Blaak et al., 2020). It has been reported that there is an interaction between the brain and intestinal tract functions and intestinal microbiota. This interrelationship is called the brain-gut-interaction or brain-gut-axis (Mayer et al., 2022). An example of the influence of the brain on intestinal function is irritable bowel syndrome, a well-known stress-related disorder. Irritable bowel syndrome is a condition in which abdominal pain and discomfort persist, with recurrent constipation and diarrhea, even in the absence of intestinal tract abnormalities. When the brain experiences anxiety or stress, the intestinal tract is hypersensitive to these signals, resulting in abnormal peristalsis, abdominal pain, diarrhea, and constipation. It has been reported that a vicious cycle occurs in which the stimulus is further transmitted to the brain, increasing distress and anxiety, thereby causing further abnormalities in peristalsis (Coss-Adame and Rao, 2014). However, basic studies using germ-free mice have been reported as an example of the influence of the gut on brain function. In germ-free mice, the response to stress is greater than that in normal mice, and the expression of brain-derived neurotrophic factor is reduced. Furthermore, transplantation of the intestinal microbiota of normal mice into germ-free mice has been shown to suppress stress response to the same level as that in normal mice (Sudo et al., 2004). In other words, it has been demonstrated that the microbiota is important for the gut-brain relationship and that fluctuations in the microbiota are linked to changes within these systems of communication (Mayer et al., 2014). Other relationships between intestinal microbiota and brain function have also been reported in relation to memory formation, cognitive function, mental health, and circadian rhythms, and clinical, epidemiological and immunological evidence suggests that the gut microbiota has a broad and significant impact on the gut-brain relationship (Dos Santos and Galiè, 2024). Furthermore, several mood disorders, such as anxiety disorders and depression, are now established to be associated with functional gastrointestinal disorders, while gastrointestinal disorders (e.g., irritable bowel syndrome, irritable bowel disease) are often associated with psychological comorbidity linked to alterations in the gut microbiota (Mangiola et al., 2016; Ancona et al., 2021). These brain-gut interactions are linked through the vagus nerve and circulatory systems (Möhle et al., 2016; de Zambotti et al., 2018; Chu et al., 2019; Sherwin et al., 2019).

Considering the interaction between intestinal bacteria and various brain functions, intestinal microbiota may also affect sleep. In fact, compared to normal mice, mice whose intestinal microbiota was removed by antibiotics showed a decrease in the duration of non-REM sleep during the inactive period, and an increase in the duration of non-REM and REM sleep during the active period. In other words, it has been reported that the sleep-wake cycle is no longer distinct (Ogawa et al., 2020). In addition, it has also been reported that when fecal transplants were performed on germ-free mice from sleep apnea model mice, the transplanted mice showed sleep disorders (Badran et al., 2020). Furthermore, there are also studies being conducted to see whether fecal transplants can improve insomnia in real world situations (Fang et al., 2023). It has also been reported that in rats, continued administration of prebiotics from the weaning period increases the diversity of intestinal microbiota in the adult period, and prevents the decrease in non-REM sleep time, even when sleep is disturbed by an electric shock (Thompson et al., 2016). In humans, feeding probiotics to adults with latent symptoms of depression, anxiety, and insomnia improved their scores on the Pittsburgh Sleep Quality Index (PSQI), along with changes in intestinal microbiota composition (Nishida et al., 2019; Chan et al., 2023; Badrfam et al., 2024; Li et al., 2024). Additionally, feeding probiotic tablets to medical students who were assumed to be chronically stressed results in a decrease in *Bifidobacterium* and an increase in *Streptococcus* and *Lachnospira*, along with improved sleep scores on the PSQI and shorter deep sleep latency (time from falling asleep to the first N3) (Nishida et al., 2019). It is also known that there is a correlation between sleep duration and the ratio of various intestinal bacteria (Shimizu et al., 2023), and between the measurement of sleep efficiency using an Actiwatch and the diversity of the intestinal microbiota and the ratio of intestinal bacteria (Smith et al., 2019). Thus, the relationship between sleep quality and gut bacteria is very close and two-way, and the gut microbiota, a highly complex microbial community, may directly or indirectly regulate the sleep-wake cycle through the microbiota-gut-brain axis (Marjot et al., 2021; Dissanayaka et al., 2024). In other words, it has been suggested that taking into account the gut microbiota may improve sleep quality.

Although there have been several reports examining the relationship between gut microbiota and sleep quality, most of them are based on subjective evaluations using questionnaires, and few discuss the relationship between sleep and gut microbiota from an EEG perspective. Furthermore, there have been no reports that have clarified their correlation or causal relationship. Therefore, with the aim of clarifying what kind of gut microbiota improves the quality of sleep, this study clarified the relationship between gut microbiota and sleep using various statistical analysis methods from a database of microbiota and sleep parameters obtained from EEG measurements during sleep.

## 2 Materials and methods

### 2.1 Study design and population

This study was conducted using data from “the comprehensive survey to establish an integrated database of food, gut microbiome, and health information (the ‘*Sukoyaka* Health Survey'),” conducted at the Hokkaido Information University. The *Sukoyaka* Health Survey is a survey of healthy Japanese men and women aged 20–80 years, excluding those with serious cerebrovascular disease, heart disease, liver disease, kidney disease, gastrointestinal disease, or infectious diseases requiring notification. The survey was conducted twice a year, in summer and winter, with the main survey being conducted in summer, and some people who were willing to participate also took part in the winter survey, and the same measurements were taken as in the summer. The *Sukoyaka* Health Survey was conducted in 2019 and 2020, as part of the Strategic Innovation Creation Program project. In this study, we analyzed data from the 2019 summer term, which included data from 642 participants in terms of attributes, body composition measurements, heart rate and EEG-derived sleep parameters, microbiota composition, and dietary survey (reference URL: https://humandbs.dbcls.jp/). The gut microbiota data from the *Sukoyaka* Health Survey was obtained by extracting DNA from stool samples collected from participants using the ISOSPIN Fecal DNA Kit (NIPPON GENE Co., Ltd., Tokyo, Japan). The extracted DNA was sequenced using the NovaSeq 6000 (Illumina, Inc., San Diego, CA, USA) to obtain high-quality read data. The classification composition was then estimated from the sequence data using Kraken2 (Wood et al., 2019). We used the data set obtained after that analysis. The EEG-based sleep assessment data from the *Sukoyaka* Health Survey was measured using HARU-1 (PGV Inc., Tokyo, Japan). HARU-1 is a portable EEG device that records brain activity via forehead-mounted dry electrodes during sleep. The recorded EEG data are processed using a proprietary algorithm to classify sleep into five stages: Wake, non-REM sleep stage 1 (N1), non-REM sleep stage 2 (N2), non-REM sleep stage 3 (N3), and REM sleep (Matsumori et al., 2022; Ueno et al., 2023). The classification of sleep stages is based on spectral analysis of EEG signals, including slow wave activity (SWA) and sleep spindle detection. HARU-1 has been validated in a small-scale study (*n* = 30) where it demonstrated a 75%−78% agreement with polysomnography (PSG) (Matsumori et al., 2022). However, it is important to note that HARU-1 does not record electromyography (EMG) or electrooculography (EOG), which are used in PSG to enhance the detection of REM sleep. Due to this limitation, its classification accuracy for REM sleep and transitions between N2 and N3 stages may be lower than that of PSG.

#### 2.1.1 Exclusion criteria

Individuals with missing values in any of the aforementioned datasets were excluded from analysis. Excluding individuals with missing values, 601 participants were included in the analysis. The average physical characteristics of the 601 participants are listed in Table 1.

**Table 1:** Physical characteristics of all subjects.

**Characteristic**	**Mean ±SE**
Age (years)	50.77 ± 0.49
Height (cm)	161.21 ± 0.33
Weight (kg)	57.14 ± 0.45
Body fat (%)	26.27 ± 0.28
BMI (kg/m^2^)	21.87 ± 0.13
Systolic blood pressure (mmHg)	116.94 ± 0.71
Diastolic blood pressure (mmHg)	77.23 ± 0.43

All value are shown as mean ± SEM. BMI, Body mass index.

### 2.2 Ethics

The Sukoyaka Health Survey was conducted in accordance with the ethical principles of the Declaration of Helsinki (revised by the World Medical Association Fortareza General Assembly in October 2013), and in compliance with the Ethical Guidelines for Medical Research for Persons (revised by the Ministry of Education, Culture, Sports, Science and Technology and the Ministry of Health, Labor, and Welfare on February 28, 2017). Written informed consent was obtained from all the participants. The Bioethics Committee of Hokkaido Information University reviewed and approved the feasibility, ethics, and scientific validity of the observational study (approval date: April 22, 2019; approval number: 2019-04).

### 2.3 Sleep parameter definitions

Sleep parameters in this study were obtained using HARU-1, a portable EEG-based sleep monitoring device. Unlike traditional PSG, HARU-1 utilizes a proprietary algorithm for sleep stage classification, and it does not strictly follow the Rechtschaffen and Kales criteria (Wolpert, 1969). Instead, sleep stages were classified based on spectral analysis of EEG signals, including SWA and sleep spindle detection (Matsumori et al., 2022). The following definitions were used for sleep parameters in this study: Sleep onset latency (SOL): The time from getting into bed to the first occurrence of any sleep stage. Wake time after sleep onset (WASO): The total duration of wakefulness after sleep onset. Sleep period time (SPT): The total time spent in all sleep stages, excluding WASO. Time to non-REM sleep stage 3 (SON3P): The time from sleep onset to the first occurrence of non-REM sleep stage 3 (N3). Stage appearance time (N1, N2, N3, and REM): The total time spent in each sleep stage during the sleep period. These values represent duration. To avoid confusion, the term “appearance time” in this study refers to the time spent in a particular sleep stage, not the latency to its onset.

### 2.4 Statistical analysis

All statistical analyses were performed using GraphPad Prism version 9.5.1 (GraphPad Software Inc., San Diego, CA, USA) and free and open source software “R” version 4.3.1 (reference URL: https://cran.r-project.org/). The flow of statistical methods for this experiment is shown in Supplementary Figure S1.

#### 2.4.1 Hierarchical cluster analysis

Hierarchical cluster analysis was performed to group subjects based on sleep parameters; SPT, WASO, SOL, SON3P, REM, N1, N2, and N3. Hierarchical cluster analysis was performed using “R.” For the hierarchical cluster analysis, the EEG-derived sleep parameter dataset was read as a csv file and used as a data frame. Next, the data frames were standardized using the “scale” function, and the standardized data frames were used for the hierarchical cluster analysis. The distance between samples was then calculated using the “dist” function included in the “stats” package. The Euclidean method was used to calculate the distance. Next, the “hclust” function included in the “stats” package was used to specify ward.D as the algorithm for cluster merger and perform the calculation. The plot function included in the “gplots” package was then used to create a tree diagram of the participants and sleep parameters, and the “heatmap.2” function was used to represent the results of the hierarchical cluster analysis in a heat map. We explored the optimal number of clusters using the elbow method, taking into account the tree diagram of the subjects and the results of the heatmap created by hierarchical cluster analysis, and determined that the number of clusters should be five, focusing on the point where the curve plot first turned sharply (Supplementary Figure S2). The participants were then classified into five clusters using the “cutree” function included in the “stat” package. The results were combined into a sleep parameters data frame with the “cbind” function. Finally, the results were output to csv format with the “write.csv” function, and the resulting csv file was used for analysis of variance (ANOVA).

#### 2.4.2 One-way ANOVA and non-parametric tests

One-way ANOVA and non-parametric tests were conducted to determine whether gut bacteria, body characteristics, and food intake differed between clusters. One-way ANOVA and non-parametric tests were performed using GraphPad Prism. The D' Agostion-Pearson test was used to assess the normality of the distributed data, whereas the Bartlett's test was used to examine whether the variation was skewed. If the data showed a normal distribution and equal variation, statistical significance was determined using the one-way ANOVA with a Tukey's *post-hoc* test. If the data showed a non-normal distribution or biased variation, statistical significance was determined using the Kruskal–Wallis test with a Dunn *post-hoc* test. Statistical significance was set at *p* < 0.05.

#### 2.4.3 Multiple regression analysis

Multiple regression analysis was conducted to determine whether there is a positive or negative relationship between gut bacteria and EEG-derived sleep parameters. The sleep parameters data and intestinal bacteria for which significant differences were found between clusters by the one-way ANOVA and non-parametric tests were extracted, and a multiple regression analysis was conducted using GraphPad Prism with the forced entry method with sleep parameters as explanatory variables, and each intestinal bacterium as objective variables. Before conducting the multiple regression analysis using GraphPad Prism, the extracted sleep parameters, intestinal bacteria, and confounding factors were converted into a. csv file as a dataset. The csv file was then read by “R” to create a data frame, the data frame was standardized by the “scale” function, the standardized data frame was output to csv format by the “write.csv” function, and the resulting csv file was used for the multiple regression analysis. Model 1 was defined as the one in which only sleep parameters were entered as explanatory variables; Model 2 was defined as the one in which age, sex, body mass index (BMI), systolic blood pressure, and diastolic blood pressure were adjusted as confounders; and Model 3 was defined as the one in which total energy intake, soluble dietary fiber intake, insoluble dietary fiber intake, sodium intake, and potassium intake were adjusted for confounders in addition to those of Model 2. Variance inflation factor (VIF) was calculated as multicollinearity, and it was confirmed that the VIF value was not >5.

#### 2.4.4 Exploratory causal analysis

A causal search using the Gaussian Bayesian network was conducted using the variables in Model 3 of the multiple regression analysis to examine the causal relationships among the variables. In this study, we wanted to create a network model that took into account the noise and uncertainty present in real data, so we used a Gaussian Bayesian network, which can flexibly infer the interactions and relationships between variables using conditional probability. We added constraints on sex and age. For example, a causal effect of gut bacteria on sex is not possible. Therefore, we constrained the node from intestinal bacteria to sex so that it would not appear. A list of the constraints is provided in Table 2. Constraints were setup using the “tiers2dblacklist” function included in the “bnlearn” package. Subsequently, sub-datasets were created from the datasets using the bootstrap method. For each subdataset, a structural learning algorithm using the hill-climbing method was employed to teach a directed acyclic graph (DAG). After recording the number and direction of nodes that appeared in the DAG and calculating the rate of appearance of the nodes relative to the entire sub-dataset, those whose rate of appearance exceeded a certain threshold were adopted as the averaged DAG. These structural learnings were performed by the “boot.strength” function included in the “bnlearn” package, and graphs were created by the “strength.plot” function. Next, parameter estimation was performed using the “bn.fit” function included in the “bnlearn” package. The maximum likelihood parameter estimation method was used as the estimation method.

**Table 2:** List of constraints on network model construction.

**No**.	**From**	**To**
1	Sex_ID	Age
2	BMI	Age
3	Systolic blood pressure	Age
4	Diastolic blood pressure	Age
5	Energy intake	Age
6	Na intake	Age
7	K intake	Age
8	Soluble fiber intake	Age
9	Insoluble fiber intake	Age
10	SOL	Age
11	SON3P	Age
12	WASO	Age
13	REM	Age
14	N1	Age
15	N2	Age
16	N3	Age
17	*Sutterella*	Age
18	*Sellimonas*	Age
19	*Odoribacter*	Age
20	*Alistipes*	Age
21	Age	Sex_ID
22	BMI	Sex_ID
23	Systolic blood pressure	Sex_ID
24	Diastolic blood pressure	Sex_ID
25	Energy intake	Sex_ID
26	Na intake	Sex_ID
27	K intake	Sex_ID
28	Soluble fiber intake	Sex_ID
29	Insoluble fiber intake	Sex_ID
30	SOL	Sex_ID
31	SON3P	Sex_ID
32	WASO	Sex_ID
33	REM	Sex_ID
34	N1	Sex_ID
35	N2	Sex_ID
36	N3	Sex_ID
37	*Sutterella*	Sex_ID
38	*Sellimonas*	Sex_ID
39	*Odoribacter*	Sex_ID
40	*Alistipes*	Sex_ID

When conducting a causal search using a Gaussian Bayesian network, this list was set up using the “tiers2dblacklist” function included in the “bnlearn” package using “R”. BMI, Body Mass Index; SOL, Sleep latency (Time taken from getting into bed to falling asleep); SON3P, Time taken from sleep onset to Non-REM sleep stage 3; WASO, wake time after sleep onset; REM, REM sleep stage appearance time; N1, Non-REM sleep stage 1 appearance time; N2, Non-REM sleep stage 2 appearance time; N3, Non-REM sleep stage 3 appearance time.

## 3 Results

### 3.1 Sleep characteristics were classified from sleep parameters using hierarchical cluster analysis

**Figure 1:**
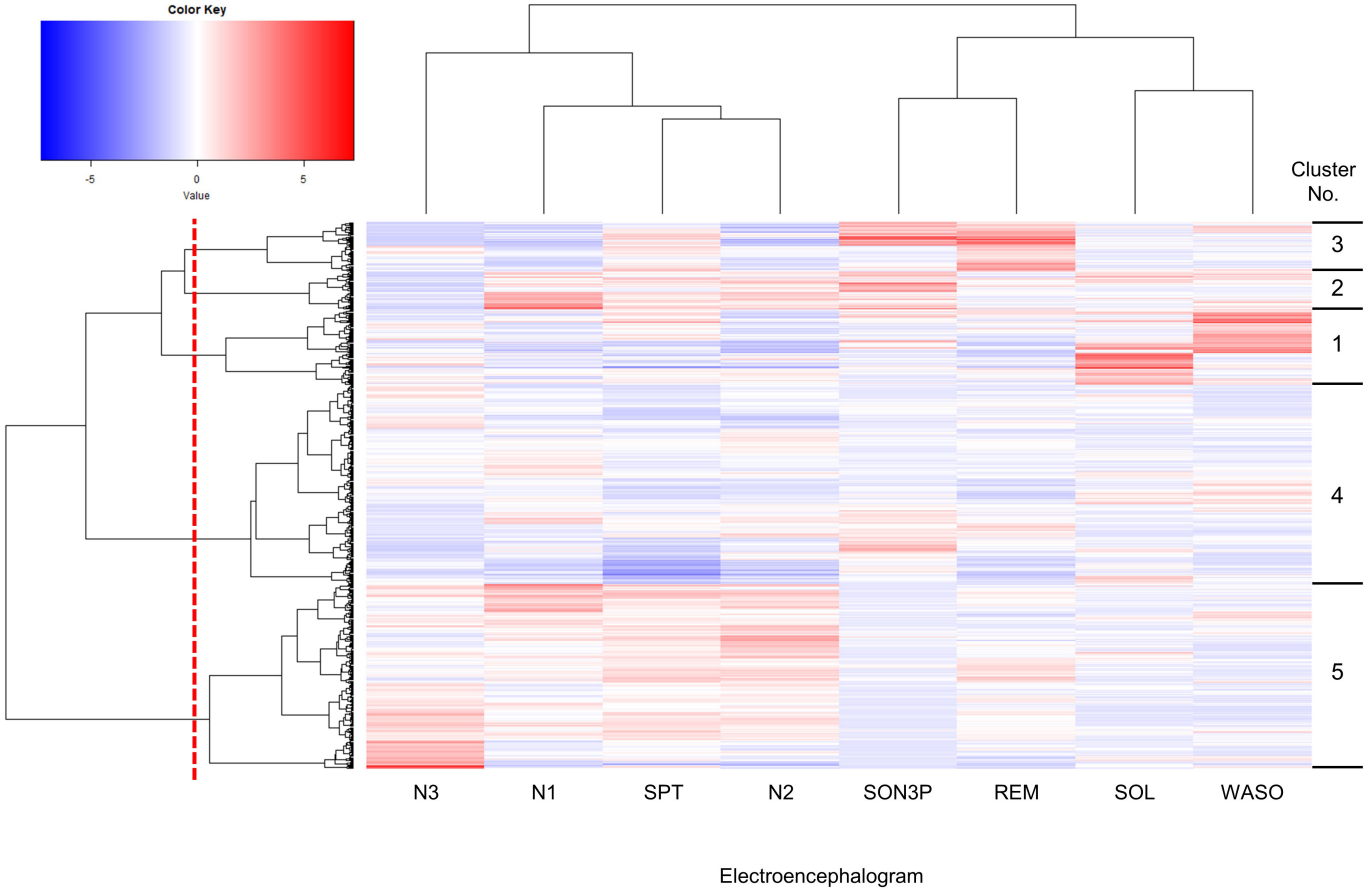
Hierarchically clustered heatmap showing the distribution of sleep parameters. Columns were hierarchical clustering with 8 sleep parameters obtained by EEG measurements, and rows were hierarchical clustering with 601 subjects. The upper cluster dendrogram is the result of clustering the sleep parameters, while the left cluster dendrogram is the result of clustering the subjects. The methods in clustering are the distance calculation method used Euclidean distance and the cluster merger algorithm was Ward.D. Sleep parameters were standardized and presented as a gradient color, with higher values indicated in red and lower values in blue. Subjects were divided into five clusters at the red dotted line shown in the cluster dendrogram of subjects, and into clusters according to sleep parameters. Cluster numbers are shown on the right-hand side of the heatmap. SPT, sleep duration; WASO, wake time after sleep onset; SOL, sleep latency (time taken from getting into bed to falling asleep); SON3P, time from sleep onset to non-REM sleep stage 3; REM, REM sleep stage appearance time; N1, non-REM sleep stage 1 appearance time; N2, non-REM sleep stage 2 appearance time; N3, non-REM sleep stage 3 appearance time.

**Figure 2:**
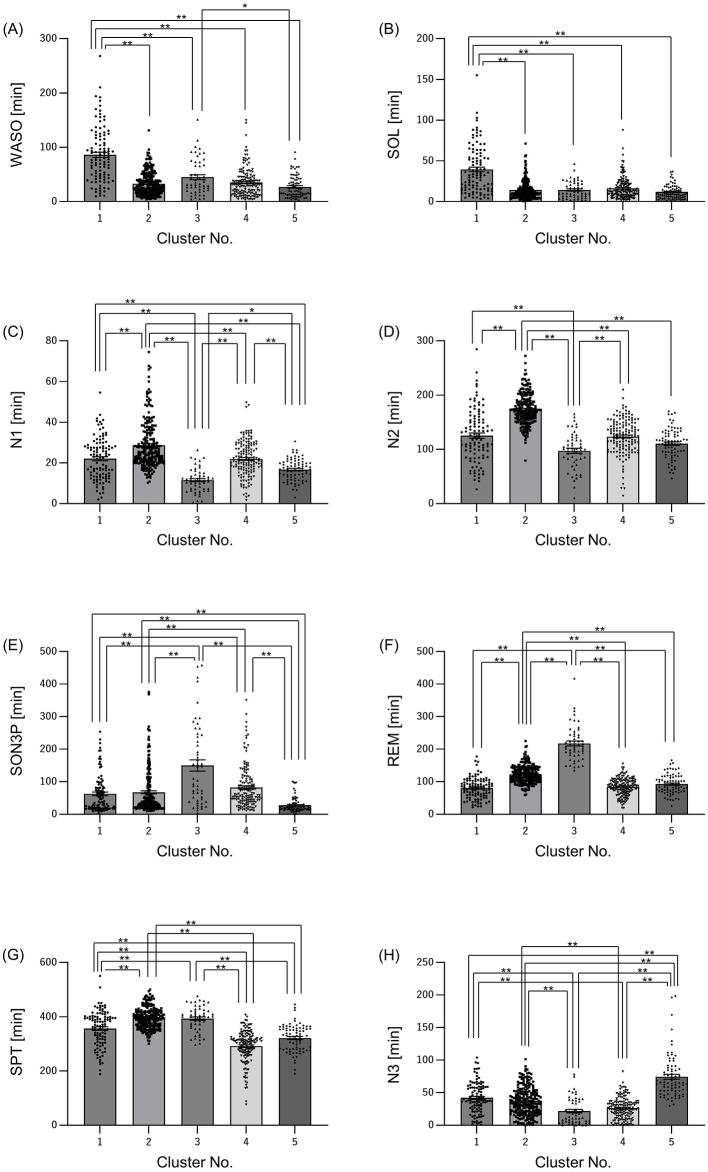
Sleep characteristics differ among clusters. Summary of sleep parameters results by cluster. **(A)** WASO: wake time after sleep onset, **(B)** SOL: sleep latency (time taken from getting into bed to falling asleep), **(C)** N1: non-REM sleep stage 1 appearance time, **(D)** N2, non-REM sleep stage 2 appearance time; **(E)** SON3P: time from sleep onset to non-REM sleep stage 3, **(F)** REM: REM sleep stage appearance time, **(G)** SPT: sleep duration, **(H)** N3: non-REM sleep stage 3 appearance time. All values are shown as mean ± standard error of the mean. **p < 0.01, *p < 0.05, evaluated using the Kruskal-Wallis test with a Dunn's *post hoc*-test.

First, we decided to group participants according to their sleep conditions, such as difficulty falling asleep or long periods of deep sleep. The participants were classified into clusters based on their EEG-derived sleep parameters (Figure 1). The sleep parameters were visualized in a heatmap, and characteristic color changes were observed between the sleep parameters. Based on these color changes and the results of searching for the optimal cluster using the elbow method, in particular, when checking the curve plot of the elbow method, the number of clusters was decided to be five because the curve first bent sharply and the within-cluster sum of squared errors was balanced at five (Supplementary Figure S2). The values of sleep parameters were tabulated for each cluster and statistical analysis was used to check whether there were significant changes between clusters and to find out the characteristics of sleep in each cluster. Cluster 1 had a significantly longer wake time after onset (WASO) and longer time spent from bedtime to falling asleep (SOL) than the other clusters (Figures 2A, B). Cluster 2 had significantly longerN1 and N2, respectively than the other clusters (Figures 2C, D). Cluster 3 had a significantly longer SON3P and longer REM sleep than the other clusters (Figures 2E, F). Cluster 4 had a significantly shorter SPT than clusters 1, 2, and 3. Cluster 4 also had the shortest sleep duration than cluster 5, although not significant, and cluster 4 had the shortest sleep duration (Figure 2G). Cluster 5 had a significantly longer N3 than the other clusters (Figure 2H). These results confirm that each cluster has different characteristics for sleep.

### 3.2 There are differences in physical characteristics and diet for each cluster

**Figure 3:**
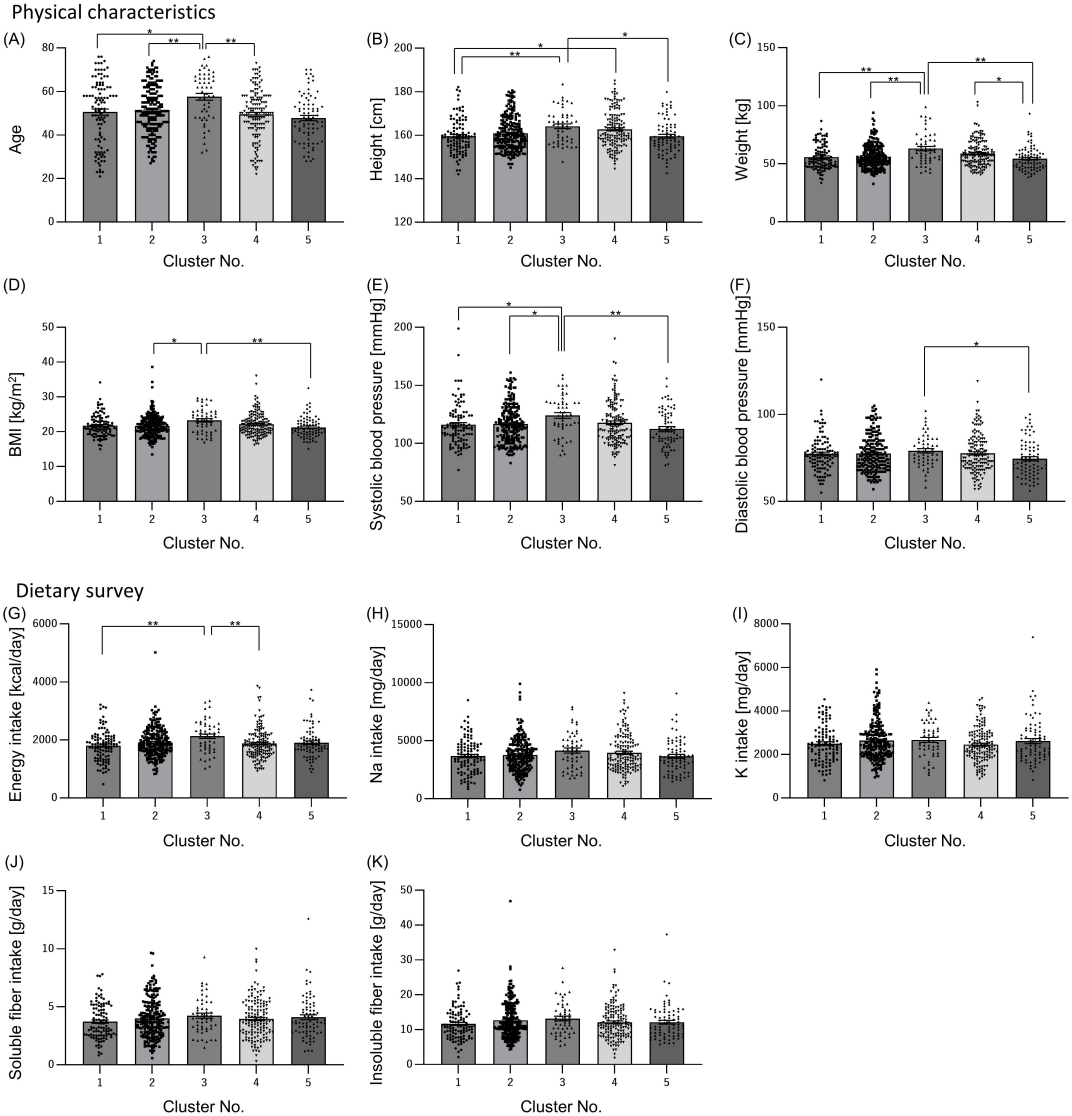
Physical characteristics and dietary survey results also differed among clusters. Physical characteristics [**(A)** Age; **(B)** Height; **(C)** Weight; **(D)** Body Mass Index (BMI); **(E)** Systolic blood pressure; **(F)** Diastolic blood pressure] and dietary survey results [**(G)** Energy intake; **(H)** Sodium intake; **(I)** Potassium intake; **(J)** Soluble dietary fiber intake; **(K)** Insoluble dietary fiber intake] were summarized by clusters. All values are shown as mean ± standard error of the mean. **p < 0.01, *p < 0.05, evaluated using the Kruska-Wallis test with a Dunn's *post hoc*-test.

Next, we examined whether there were differences in physical characteristics and dietary parameters among the clusters, as the differences in sleep characteristics and gut microbiota between the clusters could be due to physical characteristics such as age and gender. Only cluster 3 had more males than females (Table 3). Age was significantly higher in cluster 3 than that in the other clusters (Figure 3A). Height and weight were significantly higher in cluster 3 than those in clusters 1 and 5, and BMI was significantly higher in Cluster 3 than that in clusters 2 and 5 (Figures 3B–D). Systolic blood pressure was significantly higher in cluster 3 than that in clusters 1, 2, and 5, and diastolic blood pressure was significantly higher in cluster 3 than that in cluster 5 (Figures 3E, F). Energy intake was significantly higher in cluster 3 than that in clusters 1, and 4 (Figure 3G). Regarding blood pressure, which showed significant differences in physical characteristics, the intakes of sodium and potassium, which may affect blood pressure, were also examined; however, there were no significant differences among the clusters (Figures 3H, I). Additionally, the intake of soluble and insoluble fibers, which may affect the intestinal microbiota, was examined; however, there were no significant differences among the clusters (Figures 3J, K).

**Table 3:** The number of male and female subjects overall and by cluster.

**Group**	**Male**	**Female**
All subjects	196	405
Cluster_1	28	76
Cluster_2	66	155
Cluster_3	32	20
Cluster_4	50	97
Cluster_5	20	57

Numbers indicate the number of people in each.

### 3.3 Relative abundance of intestinal bacteria differ in each cluster

**Figure 4:**
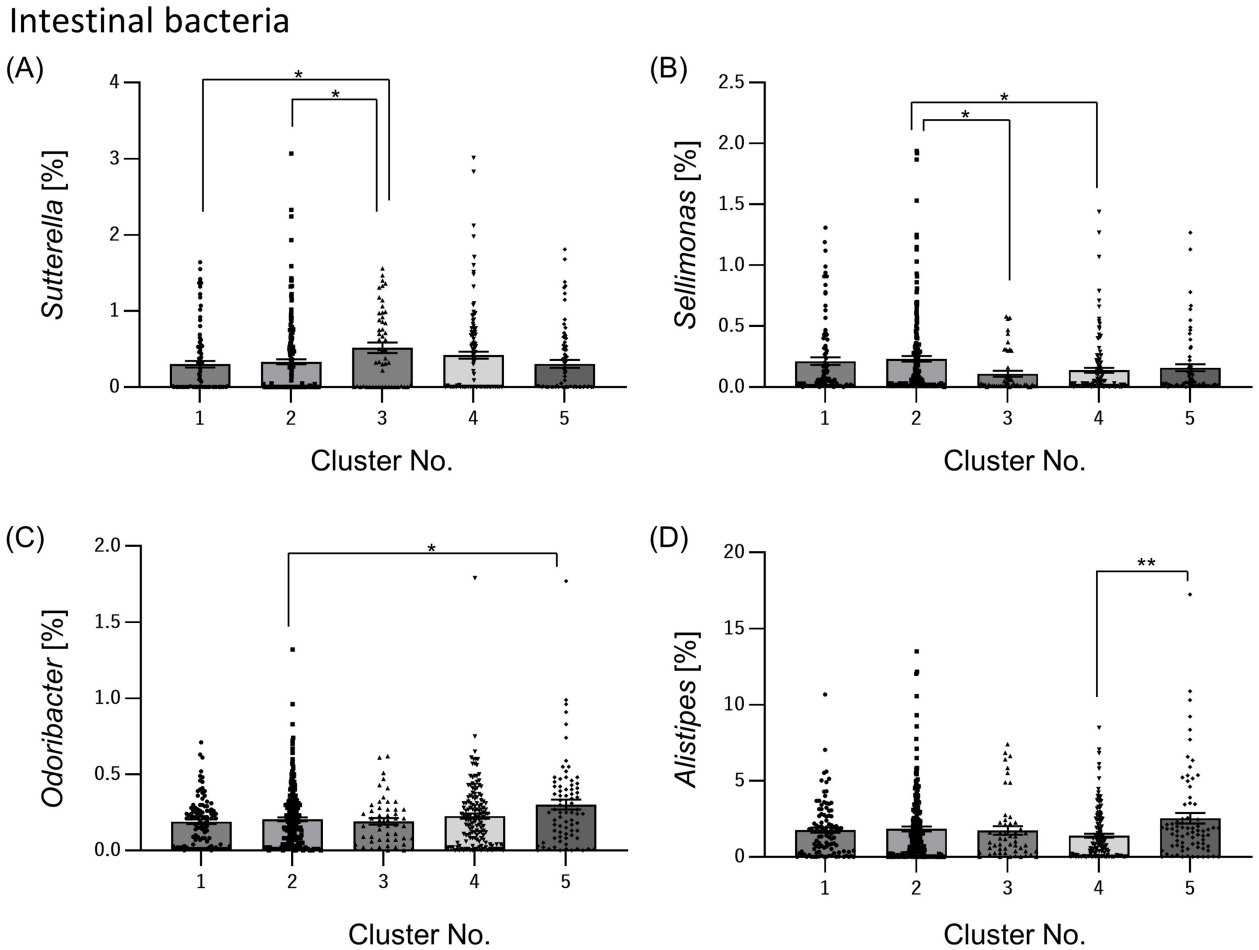
Relative abundance of intestinal bacteria differs among clusters. From the data on many intestinal bacteria, we selected the intestinal bacteria that differed significantly between clusters and summarized them by cluster [**(A)** *Sutterella*; **(B)** *Sellimonas*; **(C)** *Odoribacter*; **(D)** *Alistipes*]. All the values are shown as mean ± standard error of mean. **p < 0.01, *p < 0.05, evaluated using the Kruska-Wallis test with a Dunn's *post hoc*-test.

The relative abundance of intestinal bacteria in each cluster showed significant differences in the four genera (*Sutterella, Sellimonas, Odoribacter*, and *Alistipes*). In *Sutterella*, cluster 3 was significantly more abundant than clusters 1 and 2 (Figure 4A). Cluster 2 was significantly more abundant than clusters 3 and 4 for *Sellimonas* (Figure 4B), cluster 5 was significantly more abundant than cluster 2 for *Odoribacter* (Figure 4C), and cluster 5 was significantly more abundant than cluster 4 for *Alistipes* (Figure 4D).

### 3.4 Multiple regression analysis suggests that there is a relationship between intestinal bacteria and sleep

When the participants were clustered according to their sleep parameters, each cluster had its own unique sleep characteristics. At this point, the relative abundance of gut bacteria was calculated for each cluster, and when comparing between clusters, four types of gut bacteria were found to differ significantly between clusters. In other words, it was thought that there was a possibility that the gut microbiota would change depending on the characteristics of sleep. Therefore, in order to analyze these relationships, a regression analysis was performed, but it was shown that there were also differences in age and gender between clusters. In other words, it is possible that the characteristics of sleep and changes in the gut microbiota were affected by age, gender and diet, and that these factors could be confounding factors. For this reason, we decided to perform multiple regression analysis while taking these confounding factors into account. Multiple regression analysis was performed using each sleep parameter as an explanatory variable and gut bacteria as an objective variable. We analyzed each model as Model 1 uncorrected, Model 2 corrected for physical characteristics and Model 3 corrected for physical characteristics and diet. For the *Sutterella*, there was a significant negative association with N3 in both models (Table 4). For the *Sellimonas*, there was a significant positive association with N2 in both models (Table 5). For the *Odoribacter*, there was a significant negative association with WASO in both models (Table 6). For the *Alistipes*, there was a significant positive association between N3 and SON3P in both models (Table 7). For the *Sellimonas* and *Alistipes* in particular, there were negative and positive significant association with age in Model 2 and Model 3, respectively. These results show an association between sleep and gut bacteria, even after the modulation of physical characteristics and nutrition.

**Table 4:** Multiple regression analysis when *Sutterella* is the objective variable.

**Variables**	**Mopdel_1 (without adjustment)**	**Model_2 (adjusted for physical characteristics)**	**Model_3 (adjusted for physical characteristics and dietary survey)**
R^2^-value	0.012	0.045	0.052
SOL	−0.033 (0.455)	−0.049 (0.357)	−0.037 (0.393)
SON3P	−0.083 (0.134)	−0.068 (0.218)	−0.067 (0.224)
WASO	−0.025 (0.581)	0.003 (0.943)	−0.007 (0.878)
REM	0.0355 (0.441)	0.047 (0.314)	0.041 (0.374)
N1	0.014 (0.777)	0.017 (0.730)	0.018 (0.734)
N2	−0.116 (0.029)	−0.089 (0.100)	−0.082 (0.132)
N3	−0.149 (0.005^**^)	−0.122 (0.022^*^)	−0.120 (0.025^*^)
Age	-	−0.131 (0.004^**^)	−0.093 (0.053)
Sex	-	0.149 (0.1194)	0.094 (0.348)

The numbers are partial regression coefficients, and the numbers in parentheses are *p*-values, ^**^p < 0.01, ^*^p < 0.05. SOL, Sleep latency (Time taken from getting into bed to falling asleep); SON3P, Time taken from sleep onset to Non-REM sleep stage 3; WASO, wake time after sleep onset; REM, REM sleep stage appearance time; N1, Non-REM sleep stage 1 appearance time; N2, Non-REM sleep stage 2 appearance time; N3, Non-REM sleep stage 3 appearance time.

**Table 5:** Multiple regression analysis when *Sellimonas* is the objective variable.

**Variables**	**Mopdel_1 (without adjustment)**	**Model_2 (adjusted for physical characteristics)**	**Model_3 (adjusted for physical characteristics and dietary survey)**
R^2^-value	0.012	0.039	0.038
SOL	0.061 (0.159)	0.046 (0.282)	0.043 (0.319)
SON3P	−0.010 (0.850)	−0.005 (0.935)	−0.007 (0.906)
WASO	0.037 (0.420)	0.054 (0.237)	0.051 (0.263)
REM	−0.015 (0.747)	0.023 (0.618)	0.023 (0.623)
N1	−0.050 (0.327)	−0.023 (0.656)	−0.026 (0.623)
N2	0.170 (0.002^**^)	0.134 (0.014^*^)	0.142 (0.010^*^)
N3	0.033 (0.534)	0.011 (0.851)	0.009 (0.870)
Age	-	−0.154 (0.0007^**^)	−0.131 (0.006^**^)
Sex	-	−0.021 (0.830)	−0.009 (0.929)

The numbers are partial regression coefficients, and the numbers in parentheses are *p*-values, ^**^p < 0.01, ^*^p < 0.05. SOL, Sleep latency (Time taken from getting into bed to falling asleep); SON3P, Time taken from sleep onset to Non-REM sleep stage 3; WASO, wake time after sleep onset; REM, REM sleep stage appearance time; N1, Non-REM sleep stage 1 appearance time; N2, Non-REM sleep stage 2 appearance time; N3, Non-REM sleep stage 3 appearance time.

**Table 6:** Multiple regression analysis when *Odoribacter* is the objective variable.

**Variables**	**Mopdel_1 (without adjustment)**	**Model_2 (adjusted for physical characteristics)**	**Model_3 (adjusted for physical characteristics and dietary survey)**
R^2^-value	0.014	0.024	0.022
SOL	0.023 (0.593)	0.025 (0.563)	0.018 (0.680)
SON3P	−0.037 (0.504)	−0.044 (0.424)	−0.045 (0.416)
WASO	−0.099 (0.030^*^)	−0.117 (0.011^*^)	−0.112 (0.015^*^)
REM	−0.073 (0.115)	−0.074 (0.116)	−0.071 (0.135)
N1	−0.093 (0.067)	−0.090 (0.086)	−0.094 (0.073)
N2	−0.029 (0.588)	−0.055 (0.317)	−0.057 (0.299)
N3	0.0006 (0.989)	−0.019 (0.725)	−0.020 (0.717)
Age	-	0.050 (0.279)	0.042 (0.385)
Sex	-	−0.023 (0.813)	0.020 (0.850)

The numbers are partial regression coefficients, and the numbers in parentheses are *p*-values, ^*^p < 0.05. SOL, Sleep latency (Time taken from getting into bed to falling asleep); SON3P, Time taken from sleep onset to Non-REM sleep stage 3; WASO, wake time after sleep onset; REM, REM sleep stage appearance time; N1, Non-REM sleep stage 1 appearance time; N2, Non-REM sleep stage 2 appearance time; N3, Non-REM sleep stage 3 appearance time.

**Table 7:** Multiple regression analysis when *Alistipes* is the objective variable.

**Variables**	**Mopdel_1 (without adjustment)**	**Model_2 (adjusted for physical characteristics)**	**Model_3 (adjusted for physical characteristics and dietary survey)**
R^2^-value	0.030	0.072	0.065
SOL	0.016 (0.716)	0.022 (0.606)	0.019 (0.650)
SON3P	0.131 (0.017^*^)	0.110 (0.042^*^)	0.111 (0.042^*^)
WASO	−0.047 (0.304)	−0.077 (0.088)	−0.075 (0.097)
REM	−0.062 (0.176)	−0.074 (0.110)	−0.072 (0.118)
N1	−0.065 (0.197)	−0.081 (0.111)	−0.081 (0.113)
N2	0.081 (0.125)	0.053 (0.322)	0.053 (0.328)
N3	0.221 (< 0.0001^**^)	0.189 (0.0003^**^)	0.188 (0.0004^**^)
Age	-	0.177 (< 0.0001^**^)	0.175 (0.0002^**^)
Sex	-	−0.218 (0.021^*^)	−0.193 (0.053)

The numbers are partial regression coefficients, and the numbers in parentheses are *p-*values, ^**^p < 0.01, ^*^p < 0.05. SOL, Sleep latency (Time taken from getting into bed to falling asleep); SON3P, Time taken from sleep onset to Non-REM sleep stage 3; WASO, wake time after sleep onset; REM, REM sleep stage appearance time; N1, Non-REM sleep stage 1 appearance time; N2, Non-REM sleep stage 2 appearance time; N3, Non-REM sleep stage 3 appearance time.

### 3.5 Gaussian Bayesian network analysis shows that *Alistipes* and *Sellimonas* may affect the time of N3 and N2

**Figure 5:**
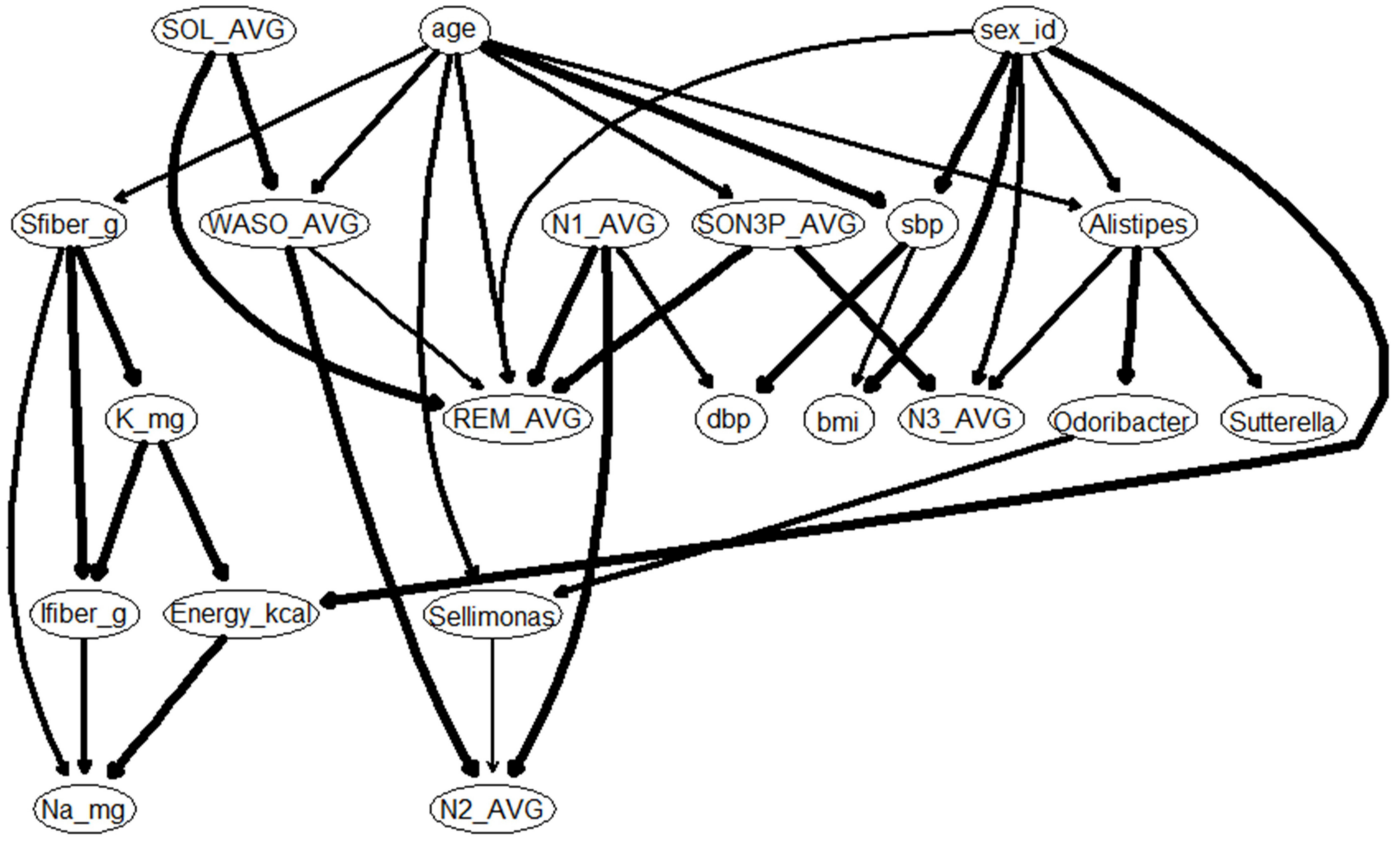
Gaussian Bayesian network model of sleep characteristics. bmi, Body Mass Index; sbp, Systolic blood pressure; dbp, Diastolic blood pressure; Sfiber_g, Soluble dietary fiber intake; Ifiber_g, Insoluble dietary fiber intake; Na_mg, Sodium intake; K_mg, Potassium intake; SOL, Sleep latency (Time taken from getting into bed to falling asleep); SON3P, Time taken from sleep onset to Non-REM sleep stage 3; WASO, wake time after sleep onset; REM, REM sleep stage appearance time; N1, Non-REM sleep stage 1 appearance time; N2, Non-REM sleep stage 2 appearance time; N3, Non-REM sleep stage 3 appearance time.

Results of multiple regression analysis found an association between sleep and gut bacteria, even after adjusting for confounding factors. However, multiple regression analysis does not reveal a causal relationship between the sleep and gut bacteria. In other words, it is not known whether sleep changed as a result of changes in gut bacteria or gut bacteria changed as a result of changes in sleep. Therefore a causal estimation was conducted to investigate the causal relationships between sleep and intestinal bacteria (Figure 5). Causal relationships between sleep and intestinal bacteria were estimated as “from *Alistipes* to N3” and “from *Sellimonas* to N2.” The coefficient for the “from *Alistipes* to N3” node was 0.113, and the coefficient for the “from *Sellimonas* to N2” node was 0.099 (Supplementary Table S1). These results suggest that participants with a higher abundance of *Alistipes* in their intestinal microbiota have more time in non-REM sleep stage 3, and may have deeper sleep, whereas those with a higher abundance of *Sellimonas* in their intestinal microbiota have more time in non-REM sleep stage 2.

## 4 Discussion

In this study, we used EEG-derived sleep parameters and gut microbiota data obtained from the *Sukoyaka* Health Study to predict these relationships using causal inference. As a result, it was predicted that an increase in *Alistipes* and *Sellimonas* would lead to an increase in non-REM sleep time, suggesting the possibility of improving sleep quality. Although there have been reports on the relationship between gut microbiota and sleep, most of them used subjective indices based on the PSQI, and few reports have evaluated this relationship using objective sleep indices. Some studies have used the Actiwatch as an objective index. However, the measurement accuracy of the Actiwatch for sleep-wake cycles, such as sleep duration, sleep onset latency, and mid-awake, is comparable to that of EEG; however, the measurement accuracy for sleep stages is still low (Liang and Chapa Martell, 2018). For example, a 2017 report conducted in collaboration with the Fitbit reported that the sleep tracker's algorithm matched PSG measurements with 70% accuracy for REM sleep and 60% for non-REM sleep, but the accuracy of measuring the deepest sleep stages decreased to approximately 50% (Beattie et al., 2017). Similarly, a 2018 report measured shallow sleep with 80% accuracy, but the accuracy decreased to approximately 50% in the deepest sleep stages (de Zambotti et al., 2018). In other words, the sleep stage results may reflect a more accurate state of sleep because EEG sleep data were obtained in this study. However, some bias may have been caused by wearing the EEG device, which would not normally be worn, and this should be considered.

Hierarchical clustering was performed using EEG-derived sleep parameters, and sleep characteristics were identified for each cluster. Consequently, the total duration spent in each sleep stage (N1, N2, N3, and REM) and the time required to transition to a sleep stage, including SOL and time to SON3P, were shown as characteristics of each cluster, and a multiple regression analysis was conducted using these values. However, for sleep quality, not only the time of appearance of sleep stages is important but also the cycle or period of sleep (Kumar, 2008). Furthermore, the time of appearance of the first non-REM sleep stage 3 may also be important for the deepest sleep stage, since growth hormone is secreted in the very first non-REM sleep stage 3 after the beginning of sleep (Kerkhofs et al., 1993). In the future, it will be necessary to analyze each sleep cycle, focusing not only on sleep duration but also on patterns of sleep stage appearance.

The percentage of non-REM sleep stage 3 is reported to be important for daytime activities, and is associated with psychiatric disorders. Deep sleep is also known to increase the risk of developing hypertension. In a follow-up study of older adult men without hypertension for approximately 3 years, 243 of 784 men developed hypertension, and only a percentage of deep sleep duration was found to be negative associated with development of hypertension (Fung et al., 2011). In this study, cluster 3, which had less time in the non-REM sleep stage 3, also had significantly higher diastolic and systolic blood pressures than cluster 5, which had more time in the non-REM sleep stage 3. In other words, the duration of non-REM sleep stage 3 may be negatively associated with higher blood pressure. The results of this study may be similar to those of previous studies. However, a causal search did not reveal a causal relationship between blood pressure and non-REM sleep stage 3. In contrast, the intestinal microbiota has also been reported to be associated with hypertension, with the composition of the intestinal microbiota being altered between healthy and hypertensive individuals (Yang et al., 2018), and transplantation of feces from hypertensive patients into germ-free mice causing an increase in blood pressure in transplanted mice (Li et al., 2017). In addition to blood pressure, type II diabetes is also known to be associated with deep sleep, and the duration of non-REM sleep stage 3 has been reported to affect insulin sensitivity, and is important for blood glucose control (Vallat et al., 2023). Furthermore, type II diabetes has also been reported to be associated with the intestinal microbiota (Cui et al., 2022; Sapp et al., 2022; Zhou et al., 2022). As this study was conducted in a wide range of healthy participants of all ages, it is possible that there was no causal relationship between blood pressure, blood glucose levels, intestinal bacteria, and sleep. Future follow-up studies over several years may show that changes in intestinal bacteria affect sleep, which may in turn improve hypertension.

A causal search was conducted, and a relationship was estimated whereby an increase in *Alistipes* caused an increase in the duration of non-REM sleep stage 3, and an increase in *Sellimonas* caused an increase in the duration of non-REM sleep stage 2. However, a relationship with age and gender was estimated for *Alistipes*, and a relationship with age was also estimated for *Sellimonas*. Furthermore, with regards to sleep, age and gender are also related to non-REM sleep stage 3 via other sleep indices. In multiple regression analysis, even after adjusting for age and sex, a significant association between *Alistipes* and non-REM sleep stage 3 and a significant association between *Sellimonas* and non-REM sleep stage 2 were found, indicating a significant association between these intestinal bacteria and sleep, regardless of age or sex. However, the effects of age and sex should not be ignored. Indeed, in multiple regression analysis, the partial regression coefficients on the adjustment factors age and gender were large and significant effects appeared. The composition of the intestinal microbiota changes with age (Odamaki et al., 2016), and it has been reported that the compositional changes and diversity of the intestinal microbiota differ, according to sex because of the influence of sex hormones (de la Cuesta-Zuluaga et al., 2019; Hatayama et al., 2023). With regard to sleep, the duration of the onset of non-REM sleep stage 3 decreases with age, and the duration of one sleep cycle becomes shorter. Studies examining sex differences in sleep have reported that difficulty falling asleep and mid-awakening are more common in women, while early morning awakenings are more common in men (Asai et al., 2006). Furthermore, in women, non-REM sleep stage 3 is known to be greatly affected by the menstrual cycle, increasing in the menstrual and follicular phases and decreasing in the early and late luteal phases (Parry et al., 1997). In consideration of the aforementioned results, this study should be interpreted with caution. In addition, the numbers of people in this study differed between men and women (Table 3), and the numbers of people in each age group differed both in total and separately for men and women (Supplementary Figure S3). Therefore, instead of performing a stratified analysis, the analysis was corrected for age and gender. In the future, it will be necessary to further increase the number of men and increase the number of people in their 20s and 60s to reduce the variation in age and gender, and then perform a stratified analysis by age and gender to improve the accuracy of the results.

There have been several studies that have looked at the relationship between subjective sleep quality and gut microbiota, for example, when comparing the gut microbiota of people with a PSQI score of 5 or more, indicating poor sleep quality, and those with a score of < 5, indicating good sleep quality the gut microbiota of people with a PSQI score of 5 or more and poor sleep quality, and those with a score of < 5 and good sleep quality, were significantly different in terms of beta diversity, and *Bacteroides, Prevotella 9*, and *Faecalibacterium* were found to be significantly associated with sleep quality (Seong et al., 2024). On the other hand, this report also reported that *Alistipes* was involved, but it reported that the relative abundance of *Alistipes* was high in humans with high PSQI scores and poor sleep quality (Seong et al., 2024), which is the exact opposite of the results of this study. In a study focusing on patients with depression and anxiety disorders, the participants were divided into an insomnia group and a non-insomnia group based on their PSQI scores. In this study, the insomnia group was found to have lower alpha diversity of the gut microbiota, such as chao1 and Shannon index (Tanaka et al., 2023). Furthermore, a positive correlation was observed between the relative abundance of the genus *Bacteroides* and PSQI scores in the non-insomnia group, indicating a potential association between the abundance of *Bacteroides* and improved sleep quality (Tanaka et al., 2023). Investigating the intestinal microbiota of patients with major depression revealed significant differences compared to healthy controls. In this study, *Dorea* was found to decrease with higher PSQI scores, suggesting a relationship between its quantity and sleep quality (Zhang et al., 2021). In previous studies, it has often been reported that low subjective sleep quality is closely related to short total sleep time and frequent middle-of-the-night awakenings as measured objectively (Åkerstedt et al., 1994, 2016; Barbato, 2021). However, on the other hand, in a study of healthy adult men and women, cluster analysis was used to group them according to the proportion of each sleep stage obtained from sleep EEG, sleep latency, number of awakenings during sleep, and total sleep time, and the groups were divided into those with good sleep quality, those with average sleep quality, and those with poor sleep quality. The study then compared subjective insomnia between the groups using the Athens Insomnia Scale, and reported that there were no significant differences between the groups (Iwagami et al., 2023). As such, it is quite possible that objective and subjective sleep indicators do not correlate with each other and may diverge, and that this divergence may be creating differences in the gut microbiota.

*Alistipes* is classified as gram-negative, rod-shaped, anaerobic, and non-spore forming. *Aristipes* is a relatively new genus of bacteria that is widely present in the gut of humans of all ethnicities and has been isolated mainly from medical clinical samples, but has a lower isolation rate than other genera in the phylum Bacteroidetes and produces acetic and propionic acids (Parker et al., 2020). *Aristipes* is largely associated with bacterial dysbiosis and disease. For example, reduced relative levels of *Aristipes* have been reported in patients with cirrhosis, metabolic dysfunction associated steatotic liver disease (MASLD) and metabolic dysfunction associated steatohepatitis (MASH), as fecal levels of acetic acid and propionic acid are reduced in MASLD and MASH, suggest that a reduction in *Aristipes* contributes to a decrease in the amount of these short-chain fatty acids and is involved in the progression of MASLD and MASH (Parker et al., 2020). Additionally, *Alistipes* have been shown to be greatly increased in mice treated with probiotics (Li et al., 2016). The relative abundance of *Alistipes* has been reported to be reduced in the gut microbiota of patients with autism spectrum disorders compared with healthy controls (Strati et al., 2017). In recent years, a comparison of the gut microbiota of healthy subjects and patients with irritable bowel syndrome has reported a decrease in the relative abundance of *Alistipes* in patients with irritable bowel syndrome (Dissanayaka et al., 2024). Other reports suggest that *Aristipes* may have preventive effects against several diseases, including liver fibrosis, colitis, cancer immunotherapy and cardiovascular disease (Parker et al., 2020). However, some reports have found a positive correlation between systolic blood pressure and *Alistipes* when comparing fecal samples from patients with hypertension and healthy controls, which may be associated with intestinal barrier dysfunction and inflammation in hypertensive patients (Parker et al., 2020). Additionally, there are reports that *Alistipes* are increased in mice placed in stressful environments (Bangsgaard Bendtsen et al., 2012), and that *Alistipes* are increased in depressed patients (Naseribafrouei et al., 2014). Although *Alistipes* can be found commonly in the intestinal tract, it has also been shown to have a significant effect on diseases with localization outside of the gut, such as depression, anxiety, chronic fatigue syndrome, autism, liver cirrhosis and aging (Parker et al., 2020). Dysbiosis within the intestine can affect the gut-brain axis and be used to explain the relationship between the gut microbiota, depression, and other mood disorders such as anxiety. It is believed that this increase in *Alistipes* disrupts the gut-brain axis because *Alistipes* is an indole-positive organism, and, thus decreases serotonin availability (Parker et al., 2020). As in these many reports, no unified conclusions have been reached on the relationship between the relative abundance of *Aristipes* and disease. However, liver cirrhosis, hypertension, autism spectrum disorders, and depression are known to be associated with sleep (Yang et al., 2018; Pandi-Perumal et al., 2020; Johnson and Zarrinnegar, 2021; Marjot et al., 2021). Therefore, the association between *Alistipes*, sleep, and these diseases requires further study. Additionally, as mentioned earlier, *Alistipes* produces acetic and propionic acids (Parker et al., 2020) and the main pathways through which these short-chain fatty acids affect the brain are the immune, endocrine, and nervous systems. In particular, as a nervous system pathway, it has been reported that short-chain fatty acids produced in the intestinal tract are absorbed via intestinal transporters and blood circulation, affect short-chain fatty acid receptors expressed in the nerve ganglia to control neural activity, and affect the brain (Westfall et al., 2017). In other words, it is possible that the short-chain fatty acids produced by *Alistipes* affect the brain and influence sleep. Sleep deprivation decreases short-chain fatty acids, suggesting a relationship between short-chain fatty acids and sleep (Shimizu et al., 2023). When tributylin, an ester composed of three molecules of butyric acid and glycerol, was administered to mice, the time spent in non-REM sleep increased by nearly 50%, indicating that butyric acid may function as a signaling molecule that induces sleep (Szentirmai et al., 2019). This suggests that the sleep-inducing effect of butyrate is mediated by sensory mechanisms in the liver and/or portal vein wall, as the increase in sleep time was observed with intra-portal administration but not with subcutaneous or intraperitoneal injection (Szentirmai et al., 2019). In addition, *Alistipes* has been reported to produce gamma-aminobutyric acid (GABA) (Vedante and Ingarao, 2024). GABA acts on enterochromaffin (EC) cells in the gut, producing serotonin, and may increase GABA in the brain via stimulation of the vagus nerve (Olsen et al., 2023; Vedante and Ingarao, 2024). Then, via GABAergic neurons, it may inhibit orexin neurons and histamine neurons that maintain wakefulness, increase delta waves, and move into a deep sleep state (Saito et al., 2018). The short-chain fatty acids mentioned above may also stimulate the vagus nerve system via the activation of G protein-coupled receptors (GPR41 and GPR43) on the intestinal mucosa (Dalile et al., 2019; Onyszkiewicz et al., 2019), and may therefore induce a deep sleep state via a pathway similar to that of GABA. As this study did not examine the metabolites of intestinal bacteria in detail, it is possible that further insights could be obtained by combining the results of this study's EEG-derived sleep parameters with data on the relative abundance of microbiota, and adding the results of bacterial metabolite analysis using metabolomics.

*Sellimonas*, a gram-positive, biased anaerobic bacterium, has been associated with a reduced risk of developing polycystic ovarian syndrome (Liang et al., 2023), and increase in gut microbiota for those with prevalent sarcopenia (Chen et al., 2023), and a higher relative abundance of *Sellimonas* is associated with a higher risk of developing breast cancer (Wei et al., 2023) and inflammatory diseases such as ulcerative colitis, and ankylosing spondylitis (Radjabzadeh et al., 2022). On the other hand, there is report that probiotic supplementation increases *Sellimonas* and improves MASLD (Hsieh et al., 2024). Although there are reports on *Sellimonas* and the disease, not many reports have examined its relationship with sleep. However, in a gut microbiota analysis of patients with ulcerative colitis, the relative abundance of *Sellimonas* was reported to be higher in ulcerative colitis patients with depression and anxiety, and in a study comparing the gut microbiota of schizophrenia patients with healthy controls and those with metabolic syndrome, *Sellimonas* was reported to be significantly higher in patients with schizophrenia (Yuan et al., 2021; Thirion et al., 2023). Significantly higher abundance of *Sellimonas* has also been reported in patients with depression (Radjabzadeh et al., 2022; Okuma et al., 2024). This suggests that *Sellimonas* may be an important bacterium in the brain-gut axis. Recently, it has been reported that *Sellimonas* is present in higher abundant in pediatric patients with autism spectrum disorders compared to healthy controls. And it was shown that fecal microbiota transplantation of healthy individuals in these patients reduced the abundance of *Sellimonas* and improved in scores on the sleep disturbance scale for children. This indicates that improving the composition of the gut microbiota, including *Sellimonas*, may have improved sleep disturbances (Dissanayaka et al., 2024). A comprehensive genomic analysis of *Sellimonas intestinalis* did not identify any genes encoding glutamate decarboxylase (GAD), an enzyme responsible for GABA synthesis, or genes involved in serotonin biosynthesis (Muñoz et al., 2020). Therefore, it is unclear whether *Sellimonas* affects sleep via GABA in the same way as *Alistipes*. However, *Sellimonas* has been associated with various inflammatory conditions and depressive symptoms, suggesting that it may indirectly affect the neurochemistry of the host (Wang et al., 2024). *Sellimonas* produces acetic acid as an end metabolite, mainly through glucose fermentation, but it is unclear how this end metabolite is associated with depression (Seo et al., 2016; Radjabzadeh et al., 2022). Further studies on *Sellimonas* spp. are needed.

It is quite possible that the quality of sleep and sleep disorders can be improved by intervening in the gut microbiota, not just in *Alistipes* and *Sellimonas*. A meta-analysis of randomized controlled trials examined whether probiotics or paraprobiotics improve sleep quality by modulating the gut microbiota. The analysis found significant improvements in sleep indices, including the PSQI, Athens Insomnia Scale, and measures of obstructive sleep apnea (OSA), leading to overall enhanced sleep quality (Yu et al., 2024). There are also reports that the intake of probiotics has an effect on non-REM sleep stage 3. When *Lactobacillus casei* Shirota was given to medical students who were feeling stressed by their exams, it was reported that *Lactobacillus casei* Shirota suppressed the decrease in non-REM sleep stage 3 sleep time as the exam date approached (Takada et al., 2017). In addition, it was also reported that the subjective indicators of “sleepiness when waking up” and “sleep length” in OSA improved. There are no reports of *Alistipes* or *Sellimonas* being given as probiotics, but in the future, research into improving sleep quality through gut bacteria will be furthered by investigating the effects of giving these bacteria as probiotics on sleep.

This study has several limitations. First, is the population; the study surveyed healthy Japanese men and women between the ages of 20–80 years, but more women than men participated in the survey, resulting in a gender bias (Table 3). Furthermore, the numbers of people in each age group differed both in total and separately for men and women, and there may also have been an age bias (Supplementary Figure S3). Additionally, the participants in the survey may have potential biases, such as better living conditions, because of their interest in health. Therefore, it is possible that the results of this survey do not fully represent the general population. Second, food and dietary data were collected on a self-reported basis. Therefore, errors and self-efficacy may have occurred with respect to the dietary data. Furthermore, although this study analyzed the amount of food consumed throughout the day, humans have a circadian clock and rhythms in food digestion, absorption, and metabolism. Therefore, the effects of eating the same food may differ, depending on the time of the day in which the food is consumed (Aoyama and Shibata, 2020). Furthermore, as well as diet composition, feeding rhythms may affect the composition of the gut cycling transcriptome and the expression of circadian rhythm genes, independently or together (Yu et al., 2024). In other words, further interpretation would be obtained if the analysis were conducted not only for the whole day but also for each meal: breakfast, lunch, and dinner. Third, this study used datasets from public databases. Those datasets do not contain sequence data such as the FASTQ files obtained from the next-generation sequencers, but only relative abundance data of gut bacteria. Therefore, it is not possible to perform alpha-diversity analysis using Simpson's diversity index or beta-diversity analysis using UniFrac distance, which can be performed from FASTQ files using analysis tools such as QIIME2. In other words, the analyses that can be carried out are limited. Thus, the analysis we can carry out is limited. Another limitation is the presence of unmeasured and uncontrolled confounding factors. Social contexts, such as economic status, marital status, and occupation, are possible confounding factors that should be considered in the future. In addition, the composition of the gut microbiota and the sleep quality are known to be affected by seasonal changes (Davenport et al., 2014; Seidler et al., 2023). Seasonal influences also need to be considered, as the survey was conducted in summer. Furthermore, this study has conducted a causal search using a Gaussian Bayesian network, but this causal search is only an estimation of the causal relationship, not a determination of it. Therefore, intervention trials would need to be conducted to determine that there is a causal relationship between *Alistipes* and *Sellimonas* and sleep quality. For example, if isolated *Aristipes* alone were transplanted into the gut of germ-free mice and the sleep conditions of the transplanted mice were altered by the transplant, these causal relationships would be further clarified. About the sleep parameters measurement, this study utilized HARU-1, a portable EEG-based sleep monitoring device, for sleep stage classification. While HARU-1 has been validated against PSG with a reported agreement of 75%−78% (Matsumori et al., 2022), the validation study was conducted with a small sample size (*n* = 30), and no large-scale replication studies have been performed. This raises concerns about the generalizability of its sleep staging accuracy to broader populations. Additionally, HARU-1 does not record EMG or EOG, which are critical for differentiating REM sleep from wakefulness and detecting muscle atonia. As a result, the classification accuracy of REM sleep and transitions between N2 and N3 may be lower than that of PSG. Furthermore, while several consumer-grade sleep trackers (e.g., Fitbit, Actiwatch) have been compared with PSG in past studies, HARU-1 has not been systematically evaluated against these devices. Future research should include comparative validation studies involving HARU-1, PSG, and other wearable sleep monitors to assess its reliability in different populations and settings.

To summarize this study, a cluster analysis was performed using EEG-derived sleep parameters results from various survey data collected from healthy men and women. The sleep characteristics were observed in each cluster, and the intestinal bacteria differed among the clusters. We predicted a causal relationship between EEG-derived sleep parameters and intestinal bacteria. The results showed a relationship in which an increase in *Alistipes* caused an increase in the duration of non-REM sleep stage 3, and an increase in *Sellimonas* caused an increase in the duration of non-REM sleep stage 2. Based on the results of this study, it may be possible to predict sleep quality by monitoring intestinal bacteria. Furthermore, controlling the intestinal bacteria may contribute to improving sleep quality. A limitation of this study is that it is only an estimation of a causal relationship and it has not been established. Therefore, future studies will be conducted to establish this causal relationship, such as transplantation of isolated bacteria into germ-free mice or intervention studies.

## Data Availability

Publicly available datasets were analyzed in this study. This data can be found here: NBDC Human database (URL: https://humandbs.dbcls.jp/en/) Research ID: hum0395.v1 JGAS000678.

## References

[B1] Åkerstedt T. Hume K. Minors D. Waterhouse J. (1994). The meaning of good sleep: a longitudinal study of polysomnography and subjective sleep quality. J. Sleep Res. 3, 152–158. 10.1111/j.1365-2869.1994.tb00122.x 10607120

[B2] Åkerstedt T. Schwarz J. Gruber G. Lindberg E. Theorell-Haglöw J. (2016). The relation between polysomnography and subjective sleep and its dependence on age - poor sleep may become good sleep. J. Sleep Res. 25, 565–570. 10.1111/jsr.12407 27122391

[B3] Ackermann S. Rasch B. (2014). Differential effects of non-REM and REM sleep on memory consolidation? Curr. Neurol. Neurosci. Rep. 14:430. 10.1007/s11910-013-0430-8 24395522

[B4] Agrawal S. Singh V. Singh C. Singh A. (2022). A review on pathophysiological aspects of Sleep Deprivation. CNS Neurol. Disord. Drug Targets 22, 1194–1208. 10.2174/1871527321666220512092718 35549867

[B5] Ancona A. Petito C. Iavarone I. Petito V. Galasso L. Leonetti A.. (2021). The gut-brain axis in irritable bowel syndrome and inflammatory bowel disease. Dig. Liver Dis. 53, 298–305. 10.1016/j.dld.2020.11.026 33303315

[B6] Aoyama S. Shibata S. (2020). Time-of-day-dependent physiological responses to meal and exercise. Front. Nutr. 7:18. 10.3389/fnut.2020.00018 32181258 PMC7059348

[B7] Asai T. Kkaneita Y. Uchiyama M. Takemura S. Asai S. Yokoyama E.. (2006). Epidemiological study of the relationship between sleep disturbances and somatic and psychological complaints among the Japanese general population. Sleep Biol. Rhythms 4, 55–62. 10.1111/j.1479-8425.2006.00197.x

[B8] Badran M. Khalyfa A. Ericsson A. Gozal D. (2020). Fecal microbiota transplantation from mice exposed to chronic intermittent hypoxia elicits sleep disturbances in naïve mice. Exp. Neurol. 334:113439. 10.1016/j.expneurol.2020.113439 32835671 PMC7642108

[B9] Badrfam R. Zandifar A. Hajialigol A. Rashidian M. Schmidt N. B. Morabito D.. (2024). Efficacy of probiotic supplements in improving the symptoms of psychosis, anxiety, insomnia, and anorexia due to amphetamine and methamphetamine use: a randomized clinical trial. Psychopharmacology 241, 1463–1476. 10.1007/s00213-024-06577-x 38512593

[B10] Bangsgaard Bendtsen K. M. Krych L. Sørensen D. B. Pang W. Nielsen D. S. Josefsen K.. (2012). Gut microbiota composition is correlated to grid floor induced stress and behavior in the BALB/c mouse. PLoS ONE 7:e46231. 10.1371/journal.pone.0046231 23056268 PMC3462757

[B11] Barbato G. (2021). REM sleep: an unknown indicator of sleep quality. Int. J. Environ. Res. Public Health 18:12976. 10.3390/ijerph182412976 34948586 PMC8702162

[B12] Beattie Z. Oyang Y. Statan A. Ghoreyshi A. Pantelopoulos A. Russell A.. (2017). Estimation of sleep stages in a healthy adult population from optical plethysmography and accelerometer signals. Physiol. Meas. 38, 1968–1979. 10.1088/1361-6579/aa9047 29087960

[B13] Blaak E. E. Canfora E. E. Theis S. Frost G. Groen A. K. Mithieux G.. (2020). Short chain fatty acids in human gut and metabolic health. Benef. Microbes 11, 411–455. 10.3920/BM2020.0057 32865024

[B14] Blanco W. Pereira C. M. Cota V. R. Souza A. C. Rennó-Costa C. Santos S.. (2015). Synaptic homeostasis and restructuring across the sleep-wake cycle. PLoS Comput. Biol. 11:e1004241. 10.1371/journal.pcbi.1004241 26020963 PMC4447375

[B15] Chan H. H. Y. Siu P. L. K. Choy C. T. Chan U. K. Zhou J. Wong C. H.. (2023). Novel multi-strain E3 probiotic formulation improved mental health symptoms and sleep quality in Hong Kong Chinese. Nutrients 15:5037. 10.3390/nu15245037 38140296 PMC10745623

[B16] Chen S. Han H. Sun X. Zhou G. Zhou Q. Li Z. (2023). Causal effects of specific gut microbiota on musculoskeletal diseases: a bidirectional two-sample Mendelian randomization study. Front. Microbiol. 14:1238800. 10.3389/fmicb.2023.1238800 37664120 PMC10469765

[B17] Chikani V. Ho K. K. (2014). Action of GH on skeletal muscle function: molecular and metabolic mechanisms. J. Mol. Endocrinol. 52, R107–123. 10.1530/JME-13-0208 24163428

[B18] Chu C. Murdock M. H. Jing D. Won T. H. Chung H. Kressel A. M.. (2019). The microbiota regulate neuronal function and fear extinction learning. Nature 574, 543–548. 10.1038/s41586-019-1644-y 31645720 PMC6818753

[B19] Coss-Adame E. Rao S. S. (2014). Brain and gut interactions in irritable bowel syndrome: new paradigms and new understandings. Curr. Gastroenterol. Rep. 16:379. 10.1007/s11894-014-0379-z 24595616 PMC4083372

[B20] Covassin N. Singh P. (2016). Sleep duration and cardiovascular disease risk: epidemiologic and experimental evidence. Sleep Med. Clin. 11, 81–89. 10.1016/j.jsmc.2015.10.007 26972035 PMC4791534

[B21] Cui J. Ramesh G. Wu M. Jensen E. T. Crago O. Bertoni A. G.. (2022). Butyrate-producing bacteria and insulin homeostasis: the microbiome and insulin longitudinal evaluation study (MILES). Diabetes 71, 2438–2446. 10.2337/db22-0168 35972231 PMC9630078

[B22] Dalile B. Van Oudenhove L. Vervliet B. Verbeke K. (2019). The role of short-chain fatty acids in microbiota-gut-brain communication. Nat. Rev. Gastroenterol. Hepatol. 16, 461–478. 10.1038/s41575-019-0157-3 31123355

[B23] Davenport E. R. Mizrahi-Man O. Michelini K. Barreiro L. B. Ober C. Gilad Y. (2014). Seasonal variation in human gut microbiome composition. PLoS ONE 9:e90731. 10.1371/journal.pone.0090731 24618913 PMC3949691

[B24] de la Cuesta-Zuluaga J. Kelley S. T. Chen Y. Escobar J. S. Mueller N. T. Ley R. E.. (2019). Age- and sex-dependent patterns of gut microbial diversity in human adults. mSystems 4:261. 10.1128/mSystems.00261-19 31098397 PMC6517691

[B25] de Zambotti M. Goldstone A. Claudatos S. Colrain I. M. Baker F. C. (2018). A validation study of Fitbit Charge 2™ compared with polysomnography in adults. Chronobiol. Int. 35, 465–476. 10.1080/07420528.2017.1413578 29235907

[B26] Dijk D. J. (2009). Regulation and functional correlates of slow wave sleep. J. Clin. Sleep Med. 5, S6–15. 10.5664/jcsm.5.2S.S6 19998869 PMC2824213

[B27] Dioufa N. Schally A. V. Chatzistamou I. Moustou E. Block N. L. Owens G. K.. (2010). Acceleration of wound healing by growth hormone-releasing hormone and its agonists. Proc. Natl. Acad. Sci. USA. 107, 18611–18615. 10.1073/pnas.1013942107 20937882 PMC2972940

[B28] Dissanayaka D. M. S. Jayasena V. Rainey-Smith S. R. Martins R. N. Fernando W. (2024). The role of diet and gut microbiota in Alzheimer's disease. Nutrients 16:412. 10.3390/nu16030412 38337696 PMC10857293

[B29] Dong Y. Li J. Zhou M. Du Y. Liu D. (2022). Cortical regulation of two-stage rapid eye movement sleep. Nat. Neurosci. 25, 1675–1682. 10.1038/s41593-022-01195-2 36396977

[B30] Dos Santos A. Galiè S. (2024). The microbiota-gut-brain axis in metabolic syndrome and sleep disorders: a systematic review. Nutrients 16:390. 10.3390/nu16030390 38337675 PMC10857497

[B31] Fang H. Yao T. Li W. Pan N. Xu H. Zhao Q.. (2023). Efficacy and safety of fecal microbiota transplantation for chronic insomnia in adults: a real world study. Front. Microbiol. 14:1299816. 10.3389/fmicb.2023.1299816 38088972 PMC10712199

[B32] Frazer M. Cabrera Y. Guthrie R. Poe G. (2021). Shining a light on the mechanisms of sleep for memory consolidation. Curr. Sleep Med. Rep. 7, 1–11. 10.1007/s40675-021-00204-3

[B33] Fung M. M. Peters K. Redline S. Ziegler M. G. Ancoli-Israel S. Barrett-Connor E.. (2011). Decreased slow wave sleep increases risk of developing hypertension in elderly men. Hypertension 58, 596–603. 10.1161/HYPERTENSIONAHA.111.174409 21876072 PMC3176739

[B34] Garbarino S. Lanteri P. Bragazzi N. L. Magnavita N. Scoditti E. (2021). Role of sleep deprivation in immune-related disease risk and outcomes. Commun. Biol. 4:1304. 10.1038/s42003-021-02825-4 34795404 PMC8602722

[B35] Genzel L. Battaglia F. (2017). “Cortico-hippocampal circuits for memory consolidation: the role of the prefrontal cortex,” in Cognitive Neuroscience of Memory Consolidation, 265–281. 10.1007/978-3-319-45066-7_16

[B36] Hatayama K. Kono K. Okuma K. Hasuko K. Masuyama H. Benno Y. (2023). Sex differences in intestinal microbiota and their association with some diseases in a japanese population observed by analysis using a large dataset. Biomedicines 11:376. 10.3390/biomedicines11020376 36830915 PMC9953495

[B37] Hori T. Sugita Y. Koga E. Shirakawa S. Inoue K. Uchida S.. (2001). Proposed supplements and amendments to 'A Manual of Standardized Terminology, Techniques and Scoring System for Sleep Stages of Human Subjects', the Rechtschaffen and Kales (1968) standard. Psychiatry Clin. Neurosci. 55, 305–310. 10.1046/j.1440-1819.2001.00810.x 11422885

[B38] Hsieh R. H. Chien Y. J. Lan W. Y. Lin Y. K. Lin Y. H. Chiang C. F.. (2024). Bacillus coagulans TCI711 supplementation improved nonalcoholic fatty liver by modulating gut microbiota: a randomized, placebo-controlled, clinical trial. Curr. Dev. Nutr. 8:102083. 10.1016/j.cdnut.2024.102083 38510931 PMC10951533

[B39] Irwin M. R. (2019). Sleep and inflammation: partners in sickness and in health. Nat. Rev. Immunol. 19, 702–715. 10.1038/s41577-019-0190-z 31289370

[B40] Iwagami M. Seol J. Hiei T. Tani A. Chiba S. Kanbayashi T.. (2023). Association between electroencephalogram-based sleep characteristics and physical health in the general adult population. Sci. Rep. 13:21545. 10.1038/s41598-023-47979-9 38066043 PMC10709300

[B41] Johnson K. P. Zarrinnegar P. (2021). Autism spectrum disorder and sleep. Child Adolesc. Psychiatr. Clin. N. Am. 30, 195–208. 10.1016/j.chc.2020.08.012 33223062

[B42] Karna B. Sankari A. Tatikonda G. (2024). “Sleep disorder,” in StatPearls (Treasure Island (FL): StatPearls Publishing). 32809555

[B43] Kerkhofs M. Van Cauter E. Van Onderbergen A. Caufriez A. Thorner M. O. Copinschi G. (1993). Sleep-promoting effects of growth hormone-releasing hormone in normal men. Am. J. Physiol. 264, E594–598. 10.1152/ajpendo.1993.264.4.E594 8476038

[B44] Koh A. De Vadder F. Kovatcheva-Datchary P. Bäckhed F. (2016). From dietary fiber to host physiology: short-chain fatty acids as key bacterial metabolites. Cell 165, 1332–1345. 10.1016/j.cell.2016.05.041 27259147

[B45] Kumar V. M. (2008). Sleep and sleep disorders. Indian J. Chest Dis. Allied Sci. 50, 129–135. 18610697

[B46] Leary E. B. Watson K. T. Ancoli-Israel S. Redline S. Yaffe K. Ravelo L. A.. (2020). Association of rapid eye movement sleep with mortality in middle-aged and older adults. JAMA Neurol. 77, 1241–1251. 10.1001/jamaneurol.2020.2108 32628261 PMC7550971

[B47] Li J. Li Y. Zhao J. Li L. Wang Y. Chen F.. (2024). Effects of Bifidobacterium breve 207-1 on regulating lifestyle behaviors and mental wellness in healthy adults based on the microbiome-gut-brain axis: a randomized, double-blind, placebo-controlled trial. Eur. J. Nutr. 63, 2567–2585. 10.1007/s00394-024-03447-2 38869657

[B48] Li J. Sung C. Y. Lee N. Ni Y. Pihlajamäki J. Panagiotou G.. (2016). Probiotics modulated gut microbiota suppresses hepatocellular carcinoma growth in mice. Proc. Natl. Acad. Sci. USA. 113, E1306–1315. 10.1073/pnas.1518189113 26884164 PMC4780612

[B49] Li J. Zhao F. Wang Y. Chen J. Tao J. Tian G.. (2017). Gut microbiota dysbiosis contributes to the development of hypertension. Microbiome 5:14. 10.1186/s40168-016-0222-x 28143587 PMC5286796

[B50] Liang Y. Zeng W. Hou T. Yang H. Wu B. Pan R.. (2023). Gut microbiome and reproductive endocrine diseases: a Mendelian randomization study. Front. Endocrinol. 14:1164186. 10.3389/fendo.2023.1164186 37600687 PMC10436605

[B51] Liang Z. Chapa Martell M. A. (2018). Validity of consumer activity wristbands and wearable eeg for measuring overall sleep parameters and sleep structure in free-living conditions. J. Healthc. Inform. Res. 2, 152–178. 10.1007/s41666-018-0013-1 35415400 PMC8982823

[B52] Lieberman H. R. Bathalon G. P. Falco C. M. Kramer F. M. Morgan C. A. 3rd, Niro, P. (2005). Severe decrements in cognition function and mood induced by sleep loss, heat, dehydration, and undernutrition during simulated combat. Biol. Psychiatry 57, 422–429. 10.1016/j.biopsych.2004.11.014 15705359

[B53] Liu J. Niethard N. Lun Y. Dimitrov S. Ehrlich I. Born J.. (2024). Slow-wave sleep drives sleep-dependent renormalization of synaptic AMPA receptor levels in the hypothalamus. PLoS Biol. 22:e3002768. 10.1371/journal.pbio.3002768 39163472 PMC11364421

[B54] Magee L. Hale L. (2012). Longitudinal associations between sleep duration and subsequent weight gain: a systematic review. Sleep Med. Rev. 16, 231–241. 10.1016/j.smrv.2011.05.005 21784678 PMC3202683

[B55] Mangiola F. Ianiro G. Franceschi F. Fagiuoli S. Gasbarrini G. Gasbarrini A. (2016). Gut microbiota in autism and mood disorders. World J. Gastroenterol. 22, 361–368. 10.3748/wjg.v22.i1.361 26755882 PMC4698498

[B56] Maquet P. Péters J. Aerts J. Delfiore G. Degueldre C. Luxen A.. (1996). Functional neuroanatomy of human rapid-eye-movement sleep and dreaming. Nature 383, 163–166. 10.1038/383163a0 8774879

[B57] Marchesi J. R. Adams D. H. Fava F. Hermes G. D. Hirschfield G. M. Hold G.. (2016). The gut microbiota and host health: a new clinical frontier. Gut 65, 330–339. 10.1136/gutjnl-2015-309990 26338727 PMC4752653

[B58] Marjot T. Ray D. W. Williams F. R. Tomlinson J. W. Armstrong M. J. (2021). Sleep and liver disease: a bidirectional relationship. Lancet Gastroenterol. Hepatol. 6, 850–863. 10.1016/S2468-1253(21)00169-2 34273289

[B59] Matsumori S. Teramoto K. Iyori H. Soda T. Yoshimoto S. Mizutani H. (2022). HARU sleep: a deep learning-based sleep scoring system with wearable sheet-type frontal EEG sensors. IEEE Access 10:3146337. 10.1109/ACCESS.2022.3146337

[B60] Mayer E. A. Nance K. Chen S. (2022). The gut-brain axis. Annu. Rev. Med. 73, 439–453. 10.1146/annurev-med-042320-014032 34669431

[B61] Mayer E. A. Savidge T. Shulman R. J. (2014). Brain-gut microbiome interactions and functional bowel disorders. Gastroenterology 146, 1500–1512. 10.1053/j.gastro.2014.02.037 24583088 PMC4114504

[B62] McCarter S. J. Hagen P. T. St Louis E. K. Rieck T. M. Haider C. R. Holmes D. R.. (2022). Physiological markers of sleep quality: a scoping review. Sleep Med. Rev. 64:101657. 10.1016/j.smrv.2022.101657 35753151

[B63] Möhle L. Mattei D. Heimesaat M. M. Bereswill S. Fischer A. Alutis M.. (2016). Ly6C(hi) monocytes provide a link between antibiotic-induced changes in gut microbiota and adult hippocampal neurogenesis. Cell Rep. 15, 1945–1956. 10.1016/j.celrep.2016.04.074 27210745

[B64] Motomura Y. Kitamura S. Oba K. Terasawa Y. Enomoto M. Katayose Y.. (2013). Sleep debt elicits negative emotional reaction through diminished amygdala-anterior cingulate functional connectivity. PLoS ONE 8:e56578. 10.1371/journal.pone.0056578 23418586 PMC3572063

[B65] Muñoz M. Guerrero-Araya E. Cortés-Tapia C. Plaza-Garrido A. Lawley T. D. Paredes-Sabja D. (2020). Comprehensive genome analyses of Sellimonas intestinalis, a potential biomarker of homeostasis gut recovery. Microb. Genom. 6:476. 10.1099/mgen.0.000476 33206037 PMC8116674

[B66] Naseribafrouei A. Hestad K. Avershina E. Sekelja M. Linløkken A. Wilson R.. (2014). Correlation between the human fecal microbiota and depression. Neurogastroenterol. Motil. 26, 1155–1162. 10.1111/nmo.12378 24888394

[B67] Nishida K. Sawada D. Kuwano Y. Tanaka H. Rokutan K. (2019). Health benefits of lactobacillus gasseri cp2305 tablets in young adults exposed to chronic stress: a randomized, double-blind, placebo-controlled study. Nutrients 11:1859. 10.3390/nu11081859 31405122 PMC6723420

[B68] Nofzinger E. A. Mintun M. A. Wiseman M. Kupfer D. J. Moore R. Y. (1997). Forebrain activation in REM sleep: an FDG PET study. Brain Res. 770, 192–201. 10.1016/S0006-8993(97)00807-X 9372219

[B69] Odamaki T. Kato K. Sugahara H. Hashikura N. Takahashi S. Xiao J. Z.. (2016). Age-related changes in gut microbiota composition from newborn to centenarian: a cross-sectional study. BMC Microbiol. 16:90. 10.1186/s12866-016-0708-5 27220822 PMC4879732

[B70] Ogawa Y. Miyoshi C. Obana N. Yajima K. Hotta-Hirashima N. Ikkyu A.. (2020). Gut microbiota depletion by chronic antibiotic treatment alters the sleep/wake architecture and sleep EEG power spectra in mice. Sci. Rep. 10:19554. 10.1038/s41598-020-76562-9 33177599 PMC7659342

[B71] Okuma K. Hatayama K. Tokuno H. Ebara A. Odachi A. Masuyama H.. (2024). A risk estimation method for depression based on the dysbiosis of intestinal microbiota in Japanese patients. Front. Psychiatry 15:1382175. 10.3389/fpsyt.2024.1382175 38863614 PMC11165696

[B72] Olsen L. K. Solis E. Jr. Mcintire L. K. Hatcher-Solis C. N. (2023). Vagus nerve stimulation: mechanisms and factors involved in memory enhancement. Front. Hum. Neurosci. 17:1152064. 10.3389/fnhum.2023.1152064 37457500 PMC10342206

[B73] Onyszkiewicz M. Gawrys-Kopczynska M. Konopelski P. Aleksandrowicz M. Sawicka A. Kozniewska E.. (2019). Butyric acid, a gut bacteria metabolite, lowers arterial blood pressure via colon-vagus nerve signaling and GPR41/43 receptors. Pflugers Arch. 471, 1441–1453. 10.1007/s00424-019-02322-y 31728701 PMC6882756

[B74] Pagani M. Lombardi F. Guzzetti S. Rimoldi O. Furlan R. Pizzinelli P.. (1986). Power spectral analysis of heart rate and arterial pressure variabilities as a marker of sympatho-vagal interaction in man and conscious dog. Circ. Res. 59, 178–193. 10.1161/01.RES.59.2.178 2874900

[B75] Pandi-Perumal S. R. Monti J. M. Burman D. Karthikeyan R. Bahammam A. S. Spence D. W.. (2020). Clarifying the role of sleep in depression: a narrative review. Psychiatry Res. 291:113239. 10.1016/j.psychres.2020.113239 32593854

[B76] Parker B. J. Wearsch P. A. Veloo A. C. M. Rodriguez-Palacios A. (2020). The genus alistipes: gut bacteria with emerging implications to inflammation, cancer, and mental health. Front. Immunol. 11:906. 10.3389/fimmu.2020.00906 32582143 PMC7296073

[B77] Parry B. L. Berga S. L. Mostofi N. Klauber M. R. Resnick A. (1997). Plasma melatonin circadian rhythms during the menstrual cycle and after light therapy in premenstrual dysphoric disorder and normal control subjects. J. Biol. Rhythms 12, 47–64. 10.1177/074873049701200107 9104690

[B78] Radjabzadeh D. Bosch J. A. Uitterlinden A. G. Zwinderman A. H. Ikram M. A. Van Meurs J. B. J.. (2022). Gut microbiome-wide association study of depressive symptoms. Nat. Commun. 13:7128. 10.1038/s41467-022-34502-3 36473852 PMC9726982

[B79] Rasmussen M. K. Mestre H. Nedergaard M. (2018). The glymphatic pathway in neurological disorders. Lancet Neurol. 17, 1016–1024. 10.1016/S1474-4422(18)30318-1 30353860 PMC6261373

[B80] Reddy O. C. van der Werf Y. D. (2020). The sleeping brain: harnessing the power of the glymphatic system through lifestyle choices. Brain Sci. 10:868. 10.3390/brainsci10110868 33212927 PMC7698404

[B81] Redwine L. Hauger R. L. Gillin J. C. Irwin M. (2000). Effects of sleep and sleep deprivation on interleukin-6, growth hormone, cortisol, and melatonin levels in humans. J. Clin. Endocrinol. Metab. 85, 3597–3603. 10.1210/jc.85.10.3597 11061508

[B82] Reutrakul S. Van Cauter E. (2018). Sleep influences on obesity, insulin resistance, and risk of type 2 diabetes. Metab. Clin. Exp. 84, 56–66. 10.1016/j.metabol.2018.02.010 29510179

[B83] Reyes-Resina I. Samer S. Kreutz M. R. Oelschlegel A. M. (2021). Molecular mechanisms of memory consolidation that operate during sleep. Front. Mol. Neurosci. 14:767384. 10.3389/fnmol.2021.767384 34867190 PMC8636908

[B84] Saito Y. C. Maejima T. Nishitani M. Hasegawa E. Yanagawa Y. Mieda M.. (2018). Monoamines inhibit GABAergic neurons in ventrolateral preoptic area that make direct synaptic connections to hypothalamic arousal neurons. J. Neurosci. 38, 6366–6378. 10.1523/JNEUROSCI.2835-17.2018 29915137 PMC6596100

[B85] Sapp P. A. Kris-Etherton P. M. Arnesen E. A. Chen See J. R. Lamendella R. Petersen K. S. (2022). Peanuts as a nighttime snack enrich butyrate-producing bacteria compared to an isocaloric lower-fat higher-carbohydrate snack in adults with elevated fasting glucose: a randomized crossover trial. Clin. Nutr. 41, 2169–2177. 10.1016/j.clnu.2022.08.004 36067589

[B86] Seidler A. Weihrich K. S. Bes F. De Zeeuw J. Kunz D. (2023). Seasonality of human sleep: polysomnographic data of a neuropsychiatric sleep clinic. Front. Neurosci. 17:1105233. 10.3389/fnins.2023.1105233 36875666 PMC9981644

[B87] Seo B. Yoo J. E. Lee Y. M. Ko G. (2016). *Sellimonas intestinalis* gen. nov., sp. nov., isolated from human faeces. Int. J. Syst. Evol. Microbiol. 66, 951–956. 10.1099/ijsem.0.000817 26637816

[B88] Seong H. J. Baek Y. Lee S. Jin H. J. (2024). Gut microbiome and metabolic pathways linked to sleep quality. Front. Microbiol. 15:1418773. 10.3389/fmicb.2024.1418773 39144221 PMC11322573

[B89] Sherwin E. Bordenstein S. R. Quinn J. L. Dinan T. G. Cryan J. F. (2019). Microbiota and the social brain. Science 366:eaar2016. 10.1126/science.aar2016 31672864

[B90] Shimizu Y. Yamamura R. Yokoi Y. Ayabe T. Ukawa S. Nakamura K.. (2023). Shorter sleep time relates to lower human defensin 5 secretion and compositional disturbance of the intestinal microbiota accompanied by decreased short-chain fatty acid production. Gut Microbes 15:2190306. 10.1080/19490976.2023.2190306 36945116 PMC10038026

[B91] Shirolapov I. Zakharov A. Smirnova D. Lyamin A. Gayduk A. (2024). The role of the glymphatic clearance system in the mechanisms of the interactions of the sleep–waking cycle and the development of neurodegenerative processes. Neurosci. Behav. Physiol. 54, 1–6. 10.1007/s11055-024-01585-y

[B92] Smith R. P. Easson C. Lyle S. M. Kapoor R. Donnelly C. P. Davidson E. J.. (2019). Gut microbiome diversity is associated with sleep physiology in humans. PLoS ONE 14:e0222394. 10.1371/journal.pone.0222394 31589627 PMC6779243

[B93] Strati F. Cavalieri D. Albanese D. De Felice C. Donati C. Hayek J.. (2017). New evidences on the altered gut microbiota in autism spectrum disorders. Microbiome 5:24. 10.1186/s40168-017-0242-1 28222761 PMC5320696

[B94] Sudo N. Chida Y. Aiba Y. Sonoda J. Oyama N. Yu X. N.. (2004). Postnatal microbial colonization programs the hypothalamic-pituitary-adrenal system for stress response in mice. J. Physiol. 558, 263–275. 10.1113/jphysiol.2004.063388 15133062 PMC1664925

[B95] Szentirmai É. Millican N. S. Massie A. R. Kapás L. (2019). Butyrate, a metabolite of intestinal bacteria, enhances sleep. Sci. Rep. 9:7035. 10.1038/s41598-019-43502-1 31065013 PMC6504874

[B96] Takada M. Nishida K. Gondo Y. Kikuchi-Hayakawa H. Ishikawa H. Suda K.. (2017). Beneficial effects of Lactobacillus casei strain Shirota on academic stress-induced sleep disturbance in healthy adults: a double-blind, randomised, placebo-controlled trial. Benef. Microbes 8, 153–162. 10.3920/BM2016.0150 28443383

[B97] Tanaka A. Sanada K. Miyaho K. Tachibana T. Kurokawa S. Ishii C.. (2023). The relationship between sleep, gut microbiota, and metabolome in patients with depression and anxiety: A secondary analysis of the observational study. PLoS ONE 18:e0296047. 10.1371/journal.pone.0296047 38117827 PMC10732403

[B98] Tavares L. Lador A. Valderrábano M. (2021). Sleep apnea and atrial fibrillation: role of the cardiac autonomic nervous system. Methodist Debakey Cardiovasc. J. 17, 49–52. 10.14797/ZYUT2951 34104320 PMC8158444

[B99] Thirion F. Speyer H. Hansen T. H. Nielsen T. Fan Y. Le Chatelier E.. (2023). Alteration of gut microbiome in patients with schizophrenia indicates links between bacterial tyrosine biosynthesis and cognitive dysfunction. Biol. Psychiatry Glob. Open Sci. 3, 283–291. 10.1016/j.bpsgos.2022.01.009 37124355 PMC10140391

[B100] Thompson R. S. Roller R. Mika A. Greenwood B. N. Knight R. Chichlowski M.. (2016). Dietary prebiotics and bioactive milk fractions improve NREM sleep, enhance REM sleep rebound and attenuate the stress-induced decrease in diurnal temperature and gut microbial alpha diversity. Front. Behav. Neurosci. 10:240. 10.3389/fnbeh.2016.00240 28119579 PMC5223485

[B101] Tobaldini E. Fiorelli E. M. Solbiati M. Costantino G. Nobili L. Montano N. (2019). Short sleep duration and cardiometabolic risk: from pathophysiology to clinical evidence. Nat. Rev. Cardiol. 16, 213–224. 10.1038/s41569-018-0109-6 30410106

[B102] Tononi G. Cirelli C. (2006). Sleep function and synaptic homeostasis. Sleep Med. Rev. 10, 49–62. 10.1016/j.smrv.2005.05.002 16376591

[B103] Tononi G. Cirelli C. (2014). Sleep and the price of plasticity: from synaptic and cellular homeostasis to memory consolidation and integration. Neuron 81, 12–34. 10.1016/j.neuron.2013.12.025 24411729 PMC3921176

[B104] Ueno K. Ishii R. Ueda M. Yuri T. Shiroma C. Hata M.. (2023). Frontal midline theta rhythm and gamma activity measured by sheet-type wearable EEG device. Front. Hum. Neurosci. 17:1145282. 10.3389/fnhum.2023.1145282 36992791 PMC10040672

[B105] Vallat R. Shah V. D. Walker M. P. (2023). Coordinated human sleeping brainwaves map peripheral body glucose homeostasis. Cell Rep. Med. 4:101100. 10.1016/j.xcrm.2023.101100 37421946 PMC10394167

[B106] Van Dongen H. P. Maislin G. Mullington J. M. Dinges D. F. (2003). The cumulative cost of additional wakefulness: dose-response effects on neurobehavioral functions and sleep physiology from chronic sleep restriction and total sleep deprivation. Sleep 26, 117–126. 10.1093/sleep/26.2.117 12683469

[B107] Vedante J. Ingarao J. (2024). The Effects of alistipes-produced GABA on the murine gut-brain serotonergic system and major depressive disorder: a research protocol. Undergr. Res. Natur. Clin. Sci. Technol. J. 8, 1–9. 10.26685/urncst.611

[B108] Veler H. (2023). Sleep and inflammation: bidirectional relationship. Sleep Med. Clin. 18, 213–218. 10.1016/j.jsmc.2023.02.003 37120163

[B109] Wang M. Song Z. Lai S. Tang F. Dou L. Yang F. (2024). Depression-associated gut microbes, metabolites and clinical trials. Front. Microbiol. 15:1292004. 10.3389/fmicb.2024.1292004 38357350 PMC10864537

[B110] Wei Z. Yang B. Tang T. Xiao Z. Ye F. Li X.. (2023). Gut microbiota and risk of five common cancers: a univariable and multivariable Mendelian randomization study. Cancer Med. 12, 10393–10405. 10.1002/cam4.5772 36880394 PMC10225193

[B111] Westfall S. Lomis N. Kahouli I. Dia S. Y. Singh S. P. Prakash S. (2017). Microbiome, probiotics and neurodegenerative diseases: deciphering the gut brain axis. Cell. Mol. Life Sci. 74, 3769–3787. 10.1007/s00018-017-2550-9 28643167 PMC11107790

[B112] Wolpert E. A. (1969). A manual of standardized terminology, techniques and scoring system for sleep stages of human subjects. Arch. Gen. Psychiatry 20, 246–247. 10.1001/archpsyc.1969.01740140118016

[B113] Wood D. E. Lu J. Langmead B. (2019). Improved metagenomic analysis with Kraken 2. Genome Biol. 20, 257. 10.1186/s13059-019-1891-0 31779668 PMC6883579

[B114] Xie L. Kang H. Xu Q. Chen M. J. Liao Y. Thiyagarajan M.. (2013). Sleep drives metabolite clearance from the adult brain. Science 342, 373–377. 10.1126/science.1241224 24136970 PMC3880190

[B115] Yang T. Richards E. M. Pepine C. J. Raizada M. K. (2018). The gut microbiota and the brain-gut-kidney axis in hypertension and chronic kidney disease. Nat. Rev. Nephrol. 14, 442–456. 10.1038/s41581-018-0018-2 29760448 PMC6385605

[B116] Yu B. Wang K. Y. Wang N. R. Zhang L. Zhang J. P. (2024). Effect of probiotics and paraprobiotics on patients with sleep disorders and sub-healthy sleep conditions: a meta-analysis of randomized controlled trials. Front. Neurol. 15:1477533. 10.3389/fneur.2024.1477533 39479010 PMC11521871

[B117] Yuan X. Chen B. Duan Z. Xia Z. Ding Y. Chen T.. (2021). Depression and anxiety in patients with active ulcerative colitis: crosstalk of gut microbiota, metabolomics and proteomics. Gut Microbes 13:1987779. 10.1080/19490976.2021.1987779 34806521 PMC8632339

[B118] Zammit G. K. Weiner J. Damato N. Sillup G. P. Mcmillan C. A. (1999). Quality of life in people with insomnia. Sleep 22, S379–385. 10394611

[B119] Zhang Q. Yun Y. An H. Zhao W. Ma T. Wang Z.. (2021). Gut microbiome composition associated with major depressive disorder and sleep quality. Front. Psychiatry 12:645045. 10.3389/fpsyt.2021.645045 34093266 PMC8175648

[B120] Zhou Z. Sun B. Yu D. Zhu C. (2022). Gut microbiota: an important player in type 2 diabetes mellitus. Front. Cell. Infect. Microbiol. 12:834485. 10.3389/fcimb.2022.834485 35242721 PMC8886906

