# Supplementary material for: Increased relative abundance of Alistipes and Sellimonas is related to stage 2 and 3 sleep duration
Source: Front Sleep. 2025 May 30;4:1478129. doi: 10.3389/frsle.2025.1478129 (PMC12713857; doi:10.3389/frsle.2025.1478129)
Supplement: Supplementary file 1 [file Presentation_1.pptx]

## Slide 1
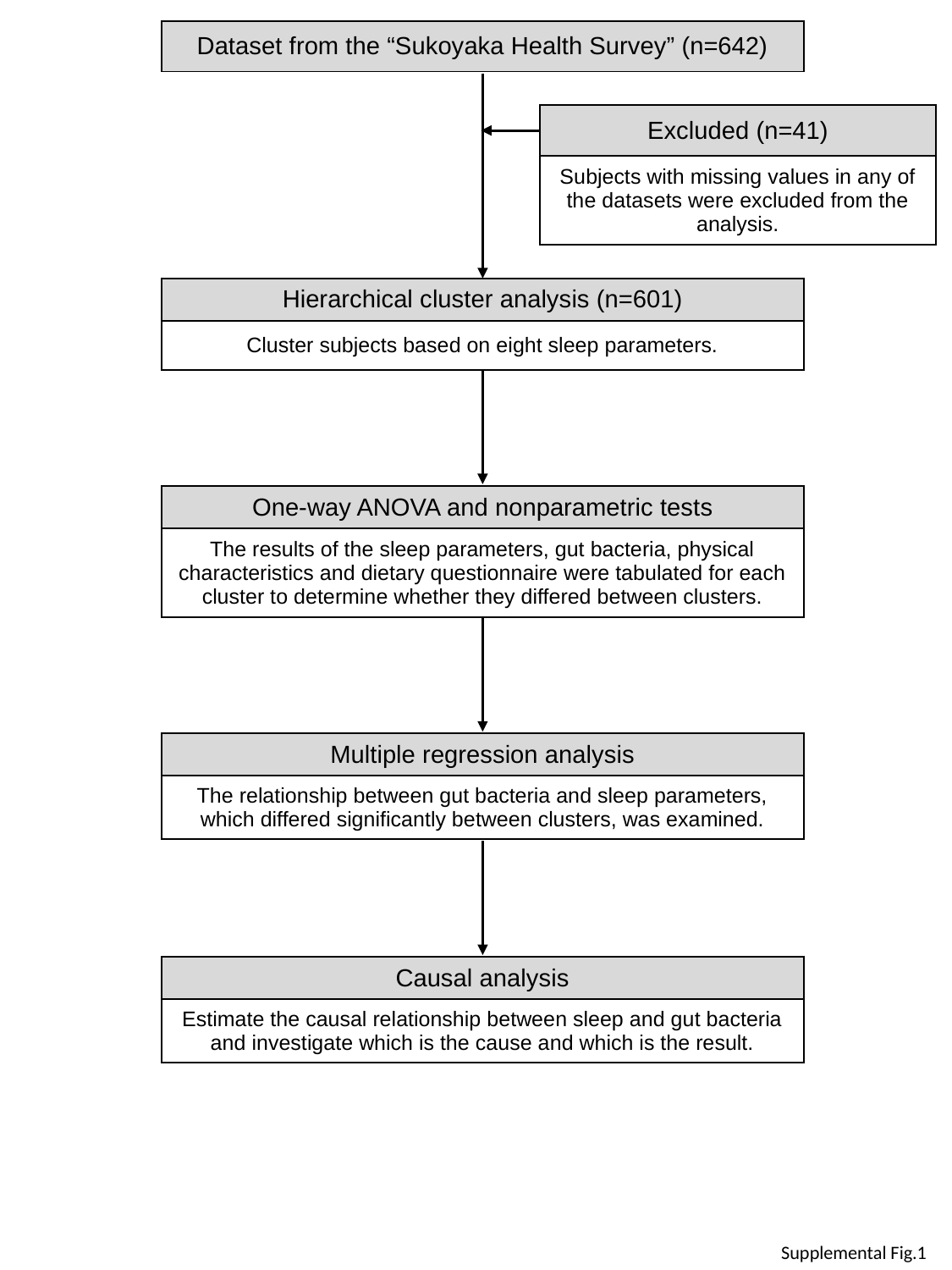

| Dataset from the “Sukoyaka Health Survey” (n=642) |
| --- |
| Excluded (n=41) |
| --- |
| Subjects with missing values in any of the datasets were excluded from the analysis. |
| Hierarchical cluster analysis (n=601) |
| --- |
| Cluster subjects based on eight sleep parameters. |
| One-way ANOVA and nonparametric tests |
| --- |
| The results of the sleep parameters, gut bacteria, physical characteristics and dietary questionnaire were tabulated for each cluster to determine whether they differed between clusters. |
| Multiple regression analysis |
| --- |
| The relationship between gut bacteria and sleep parameters, which differed significantly between clusters, was examined. |
| Causal analysis |
| --- |
| Estimate the causal relationship between sleep and gut bacteria and investigate which is the cause and which is the result. |
Supplemental Fig.1

## Slide 2
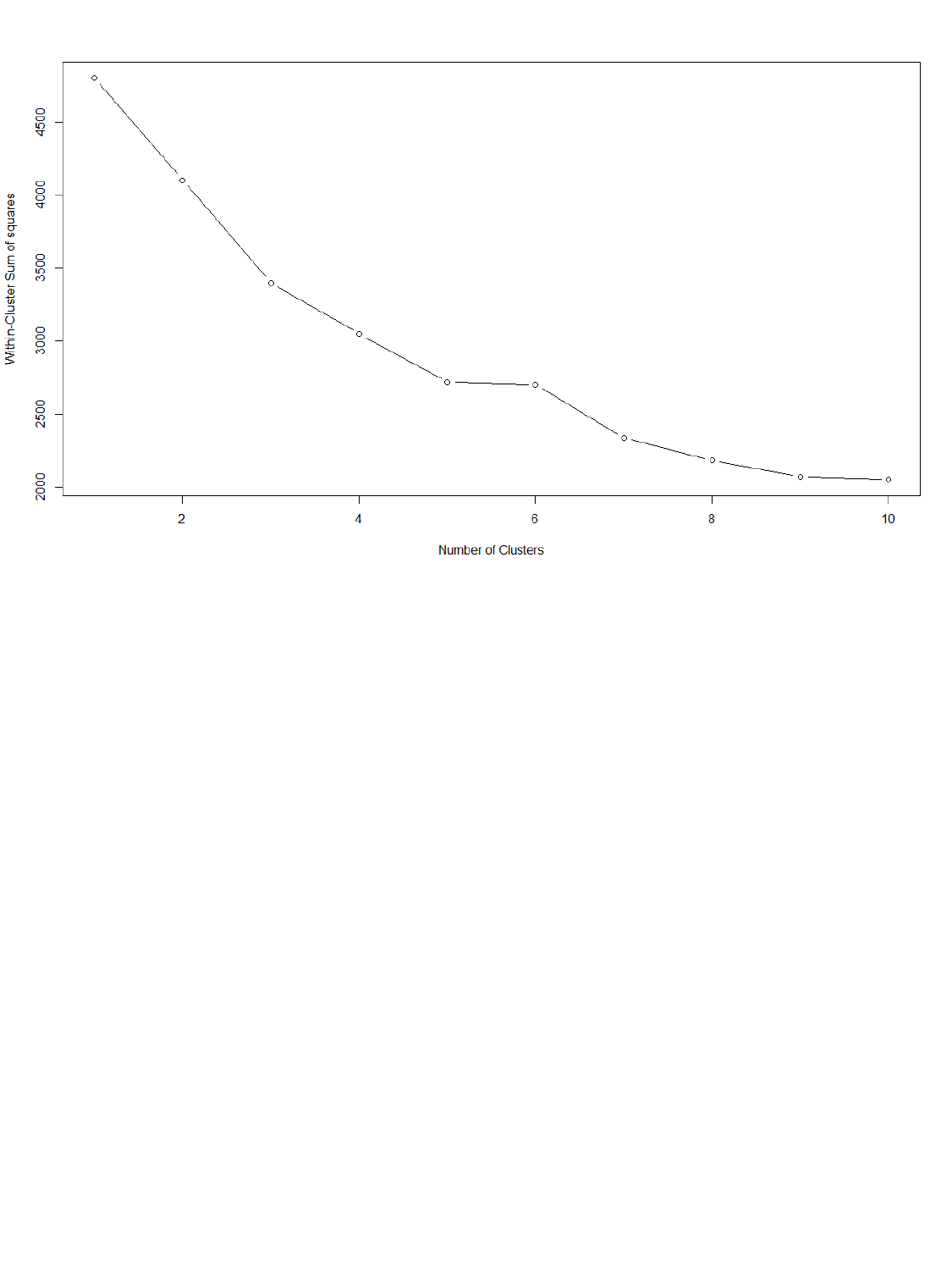

## Slide 3
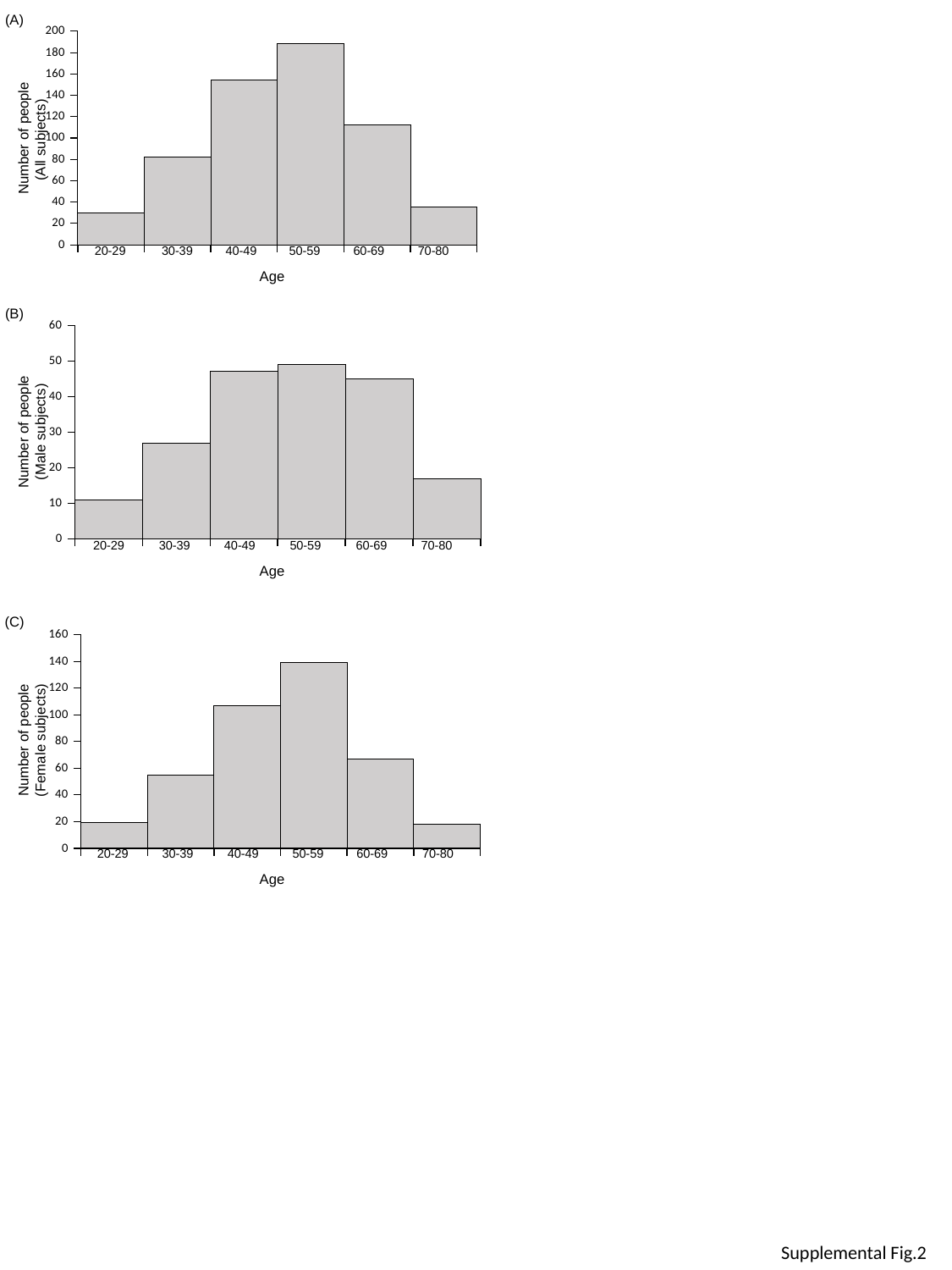

(A)
### Chart
| Category | |
|---|---|
| 29 | 30.0 |
| 39 | 82.0 |
| 49 | 154.0 |
| 59 | 188.0 |
| 69 | 112.0 |
| 79 | 35.0 |Number of people
(All subjects)
20-29
30-39
40-49
50-59
60-69
70-80
Age
(B)
### Chart
| Category | |
|---|---|
| 29 | 11.0 |
| 39 | 27.0 |
| 49 | 47.0 |
| 59 | 49.0 |
| 69 | 45.0 |
| 79 | 17.0 |Number of people
(Male subjects)
20-29
30-39
40-49
50-59
60-69
70-80
Age
(C)
### Chart
| Category | |
|---|---|
| 29 | 19.0 |
| 39 | 55.0 |
| 49 | 107.0 |
| 59 | 139.0 |
| 69 | 67.0 |
| 79 | 18.0 |Number of people
(Female subjects)
20-29
30-39
40-49
50-59
60-69
70-80
Age
Supplemental Fig.2
