# Supplementary material for: Increased relative abundance of Alistipes and Sellimonas is related to stage 2 and 3 sleep duration
Source: Front Sleep. 2025 May 30;4:1478129. doi: 10.3389/frsle.2025.1478129 (PMC12713857; doi:10.3389/frsle.2025.1478129)
Supplement: Supplementary file 2 [file Data_Sheet_1.docx]

Supplementary Material

# Supplementary Figure legend

## Supplemental Figure 1

Flow diagram showing the process of statistical analysis used in this study after the dataset was first obtained.

## Supplemental Figure 2

The curve plots of elbow criterion technique for hierarchically clustered analysis in Figure 1

## Supplemental Figure 3

The horizontal axis indicate the age and the vertical axis indicate the number of people. (A) All subjects (n=601). (B) Male subjects (n=196). (C) Female subjects (n=405).

**Supplemental Table 1. List of parameters in the constructed network model**

|  | Explanatory variable | | | | | | | | | | | | | | | | | | | | | |
| --- | --- | --- | --- | --- | --- | --- | --- | --- | --- | --- | --- | --- | --- | --- | --- | --- | --- | --- | --- | --- | --- | --- |
| Object Variable |  | Age | Sex_ID | BMI | SBP | DBP | Energy | Na | K | Soluble fiber | Insoluble fiber | SOL | SON3P | WASO | REM | N1 | N2 | N3 | Sutterella | Sellimonas | Odoribacter | Alistipes |
|  | Age |  |  |  |  |  |  |  |  |  |  |  |  |  |  |  |  |  |  |  |  |  |
|  | Sex_ID |  |  |  |  |  |  |  |  |  |  |  |  |  |  |  |  |  |  |  |  |  |
|  | BMI |  | -0.235 |  | 0.271 |  |  |  |  |  |  |  |  |  |  |  |  |  |  |  |  |  |
|  | SBP | 0.362 | -0.266 |  |  |  |  |  |  |  |  |  |  |  |  |  |  |  |  |  |  |  |
|  | DBP |  |  |  | 0.843 |  |  |  |  |  |  |  |  |  |  | 0.072 |  |  |  |  |  |  |
|  | Energy |  | -0.305 |  |  |  |  |  | 0.478 |  |  |  |  |  |  |  |  |  |  |  |  |  |
|  | Na |  |  |  |  |  | 0.458 |  |  | 0.307 | -0.207 |  |  |  |  |  |  |  |  |  |  |  |
|  | K |  |  |  |  |  |  |  |  | 0.708 |  |  |  |  |  |  |  |  |  |  |  |  |
|  | Soluble fiber | 0.253 |  |  |  |  |  |  |  |  |  |  |  |  |  |  |  |  |  |  |  |  |
|  | Insoluble fiber |  |  |  |  |  |  |  | 0.294 | 0.600 |  |  |  |  |  |  |  |  |  |  |  |  |
|  | SOL |  |  |  |  |  |  |  |  |  |  |  |  |  |  |  |  |  |  |  |  |  |
|  | SON3P | 0.184 |  |  |  |  |  |  |  |  |  |  |  |  |  |  |  |  |  |  |  |  |
|  | WASO | 0.168 |  |  |  |  |  |  |  |  |  | 0.286 |  |  |  |  |  |  |  |  |  |  |
|  | REM | 0.162 | -0.113 |  |  |  |  |  |  |  |  | -0.183 | 0.334 | -0.158 |  | -0.176 |  |  |  |  |  |  |
|  | N1 |  |  |  |  |  |  |  |  |  |  |  |  |  |  |  |  |  |  |  |  |  |
|  | N2 |  |  |  |  |  |  |  |  |  |  |  |  | -0.310 |  | 0.546 |  |  |  | 0.099 |  |  |
|  | N3 |  | 0.120 |  |  |  |  |  |  |  |  |  | -0.606 |  |  |  |  |  |  |  |  | 0.113 |
|  | Sutterella |  |  |  |  |  |  |  |  |  |  |  |  |  |  |  |  |  |  |  |  | -0.167 |
|  | Selimonas | -0.172 |  |  |  |  |  |  |  |  |  |  |  |  |  |  |  |  |  |  | -0.121 |  |
|  | Odoribacter |  |  |  |  |  |  |  |  |  |  |  |  |  |  |  |  |  |  |  |  | 0.389 |
|  | Alistipes | 0.113 | 0.184 |  |  |  |  |  |  |  |  |  |  |  |  |  |  |  |  |  |  |  |

For each node of the network model shown in Figure 5, parameter estimation was performed using the "bn.fit" function included in the "bnlearn" package. The maximum likelihood parameter estimation method was used.

BMI: Body mass index. SBP: Systolic blood pressure. DBP: Diastolic blood pressure. SOL: Sleep latency (Time taken from getting into bed to falling asleep). SON3P: Time taken from sleep onset to Non-REM sleep stage 3. WASO: wake time after sleep onset. REM: REM sleep stage appearance time. N1: Non-REM sleep stage 1 appearance time. N2: Non-REM sleep stage 2 appearance time. N3: Non-REM sleep stage 3 appearance time.
